# Discovery of New 3,3-Diethylazetidine-2,4-dione Based Thiazoles as Nanomolar Human Neutrophil Elastase Inhibitors with Broad-Spectrum Antiproliferative Activity

**DOI:** 10.3390/ijms23147566

**Published:** 2022-07-08

**Authors:** Beata Donarska, Marta Świtalska, Joanna Wietrzyk, Wojciech Płaziński, Magdalena Mizerska-Kowalska, Barbara Zdzisińska, Krzysztof Z. Łączkowski

**Affiliations:** 1 Department of Chemical Technology and Pharmaceuticals, Faculty of Pharmacy, Collegium Medicum, Nicolaus Copernicus University, Jurasza 2, 85-089 Bydgoszcz, Poland; 265000@stud.umk.pl; 2 Hirszfeld Institute of Immunology and Experimental Therapy, Polish Academy of Sciences, Rudolfa Weigla 12, 53-114 Wrocław, Poland; marta.switalska@hirszfeld.pl (M.Ś.); joanna.wietrzyk@hirszfeld.pl (J.W.); 3 Jerzy Haber Institute of Catalysis and Surface Chemistry, Polish Academy of Sciences, Niezapominajek 8, 30-239 Cracow, Poland; wojtek_plazinski@tlen.pl; 4 Department of Biopharmacy, Medical University of Lublin, Chodzki 4a, 20-093 Lublin, Poland; 5 Department of Virology and Immunology, Faculty of Biology and Biotechnology, Maria Curie-Skłodowska University, Akademicka 19 Street, 20-033 Lublin, Poland; magdalena.mizerska-kowalska@mail.umcs.pl (M.M.-K.); basiaz@poczta.umcs.lublin.pl (B.Z.)

**Keywords:** human neutrophil elastase, antiproliferative activity, thiazole, molecular docking

## Abstract

A series of 3,3-diethylazetidine-2,4-dione based thiazoles **3a**–**3j** were designed and synthesized as new human neutrophil elastase (HNE) inhibitors in nanomolar range. The representative compounds **3c**, **3e**, and **3h** exhibit high HNE inhibitory activity with IC_50_ values of 35.02–44.59 nM, with mixed mechanism of action. Additionally, the most active compounds **3c** and **3e** demonstrate high stability under physiological conditions. The molecular docking study showed good correlation of the binding energies with the IC_50_ values, suggesting that the inhibition properties are largely dependent on the stage of ligand alignment in the binding cavity. The inhibition properties are correlated with the energy level of substrates of the reaction of ligand with Ser195. Moreover, most compounds showed high and broad-spectrum antiproliferative activity against human leukemia (MV4-11), human lung carcinoma (A549), human breast adenocarcinoma (MDA-MB-231), and urinary bladder carcinoma (UMUC-3), with IC_50_ values of 4.59–9.86 μM. Additionally, compounds **3c** and **3e** can induce cell cycle arrest at the G2/M phase and apoptosis via caspase-3 activation, leading to inhibition of A549 cell proliferation. These findings suggest that these new types of drugs could be used to treat cancer and other diseases in which immunoreactive HNE is produced.

## 1. Introduction

Human neutrophil elastase (HNE, E.C. 3.4.21.37) is a 30 kDa serine protease of the chymotrypsin family stored in the azurophilic granules of neutrophils. This enzyme can hydrolyse extracellular matrix proteins such as elastin, fibronectin, proteoglycans, and collagens. Due to the fact that HNE is the main mediator of inflammation, it participates in the pathogenesis of various inflammatory diseases, including acute lung injury (ALI), acute respiratory distress syndrome (ARDS), chronic obstructive pulmonary disease (COPD), and rheumatoid arthritis and Wegener’s granulomatosis [1,2,3,4,5]. Due to the possibility of tissue damage, HNE activity is regulated by specific endogenous inhibitors such as α1-antitrypsin, α2-macroglobulin, elafin and secretory leukocyte protease inhibitor [6,7,8]. Human neutrophil elastase has also been associated with the metastasis and progression of various types of cancer via its degradation of extracellular matrix components. A high immunoreactivity of HNE in patients with breast cancer and lung cancer is correlated with poor prognosis and a reduction in the survival rate [9,10,11,12,13,14,15,16]. HNE is responsible for cellular proliferation of lung cancer cells [17]. 

**Figure 1:**
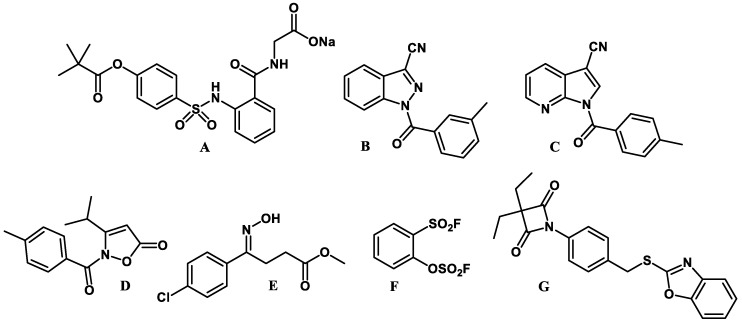
Structures of selected HNE inhibitors.

Therefore, human neutrophil elastase has become an important therapeutic target, which has resulted in the intensive search for small, effective HNE inhibitors as promising therapeutics. In recent years, many HNE inhibitors belonging to different classes of compounds have been designed (Figure 1), such as sivelestat sodium hydrate A (ONO-5046, Elaspol) [18], *N*-benzoylindazoles B [19], indoles [20], 1*H*-pyrrolo [2,3-b]pyridines C [21], isoxazol-5(2*H*)-ones D [22], heteroaryl oxime esters E [23], sulfonyl fluorides (SuFEx) F [24], and phthalimides [25]. A very interesting group of compounds that caught our attention are azetidine-2,4-diones, also known as 4-oxo-β-lactams [26]. These compounds showed the ability to serine acylation in HNE, and further research on the structure optimization showed that *N*-phenyl derivatives of 3,3-diethylazetidine-2,4-dione G (Figure 1) show much higher activity and selectivity towards HNE [27,28,29,30,31,32]. 

Due to the broad spectrum of activity, the thiazole scaffold is currently one of the most studied heterocyclic compounds [33,34]. We previously reported that compounds containing the thiazole ring show high antitumor activity and are characterized by low toxicity to healthy cells [35,36,37].

The aim of our project was two-fold. Firstly, we aimed at designing hybrid molecules containing the 3,3-diethylazetidine-2,4-dione and thiazole systems, which have the ability to inhibit human neutrophil elastase. Secondly, we investigated whether such derivatives exhibit anticancer activity. In addition, through the introduction of different substituents, we modified the electronic properties and geometry of the molecules. The inhibition of human neutrophil elastase was carried out by the spectrofluorimetric method using N-methoxysuccinyl-Ala-Ala-Pro-Val-7-amido-4-methylcoumarin (MeOSuc-AAPV-AMC) as substrate. The antiproliferative activity was tested against four human cancer cell lines, leukemia (MV4-11), lung (A549), breast (MDA-MB-231), urinary bladder (UMUC-3), and normal mouse fibroblast (BALB/3T3) cells using the 3-(4,5-dimethylthiazol-2-yl)-2,5-diphenyltetrazoliun bromide (MTT) or sulforhodamine B (SRB) assays. The molecular docking study of the inhibitors to the HNE binding site was used to analyze the influence of pharmacophore group on the activity of the studied compounds.

## 2. Results and Discussion

### 2.1. Chemical Synthesis

**Scheme 1:**
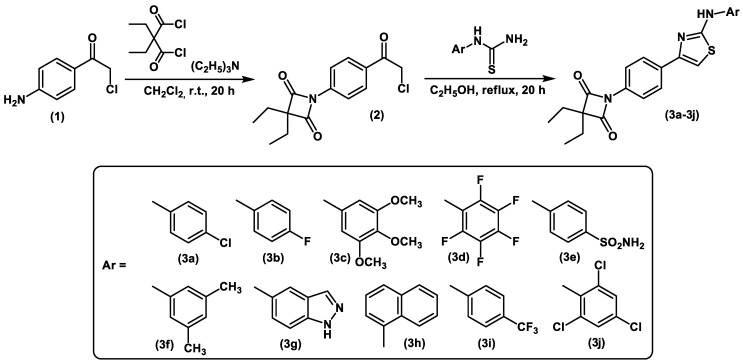
Synthesis of the 3,3-diethylazetidine-2,4-dione based thiazoles **3a**–**3j**.

The synthetic pathway leading to the final thiazole derivatives **3a**–**3j** is reported in Scheme 1. In the first step 1-(4-(2-chloroacetyl)phenyl)-3,3-diethylazetidine-2,4-dione (**2**) was obtained starting from previously described 1-(4-aminophenyl)-2-chloroethanone (**1**) [38] treated with the appropriate 2,2-diethyl malonyl dichloride and triethylamine in dry dichloromethane. Next, using the Hantzsch reaction of precursor **2** with ten different thioureas, appropriate thiazole derivatives **3a**–**3j** were obtained with 52–99% yields. The structure of all compounds was confirmed on the basis of spectral data, including ^1^H NMR (400 MHz), ^13^C NMR (100 MHz), and ESI-HRMS analysis (see Supplementary material). The ^1^H NMR spectrum of precursor 2 showed characteristic signals derived from two ethyl and a chloroacetyl groups at 0.98, 1.86 and 5.19 ppm, respectively. There is also a characteristic signal from the carbonyl group of the chloroacetyl fragment in the ^13^C NMR spectrum at about 191 ppm. The final thiazoles **3a**–**3j** showed characteristic singlets at (7.30–7.51) ppm derived from thiazole-5*H* proton, whereas in the ^13^C NMR spectrum we observe characteristic peaks at about 9.40 and 23.30 ppm, and at about 172 ppm derived from the 3,3-diethylazetidine-2,4-dione system. The high resolution mass spectrometry spectra fully support the proposed structures of the target compounds.

### 2.2. Elastase Inhibitory Activity and Kinetic Analysis

Inhibition of human neutrophil elastase was carried out by the spectrofluorimetric method using N-methoxysuccinyl-Ala-Ala-Pro-Val-7-amido-4-methyl-coumarin (MeOSuc-AAPV-AMC) as a substrate, and compared with sivelestat, which is a selective inhibitor of human neutrophil elastase. The results are summarized in Table 1. Curves for the determination of IC_50_ values for inhibition of HNE activity of compounds **3a**–**3j** and sivelestat can be found in Supporting Information (see Figures S1–S11). All tested compounds **3a**–**3j** showed the ability to inhibit HNE in nanomolar concentration, with IC_50_ values in the range of 35.02–312.19 nM. Among the tested compounds, the highest activity was demonstrated by thiazoles **3c**, **3e** and **3h** with IC_50_ values 38.25, 35.02 and 44.59 nM, respectively. These compounds contain the trimethoxyphenyl, benzenesulfonamide and naphthyl groups. These values are only two times lower than the inhibition value for standard sivelestat, which was 18.78 nM. It can be seen that the HNE inhibitory activity increases in a series of compounds **3b**, **3i** and **3d** containing 4-fluorophenyl, 4-trifluoromethylphenyl and perfluorophenyl groups with IC_50_ values 176.28, 167.57, and 110.02 nM, respectively. This is probably due to an increased electron-acceptor or steric effect. The opposite effect is observed for compounds **3a** and **3j** containing the 4-chlorophenyl and 2,4,6-trichlorophenyl groups with IC_50_ 227.29 and 248.74 nM, respectively. Also, the same relationship can be seen for derivatives **3f** and **3j** containing the 3,5-dimethylphenyl and 2,4,6-trichlorophenyl groups with IC_50_ values of 243.96 and 248.74 nM, respectively.

Compound **3g** containing the indazole group shows the lowest inhibitory activity, IC_50_ 312.19 nM, and replacement of this group with a naphthyl group significantly increases the inhibitory activity. 

Next, kinetic analysis of the mechanism of inhibition of human neutrophil elastase by thiazoles **3a**–**3j** was determined using double-reciprocal plots of Lineweaver-Burk plots and Dixon analysis. The results are shown in Table 1. In our research, we showed that all derivatives **3a**–**3j** showed a mixed mechanism of inhibition of elastase, indicating that the tested compounds can inhibit elastase in two different models.

**Figure 2:**
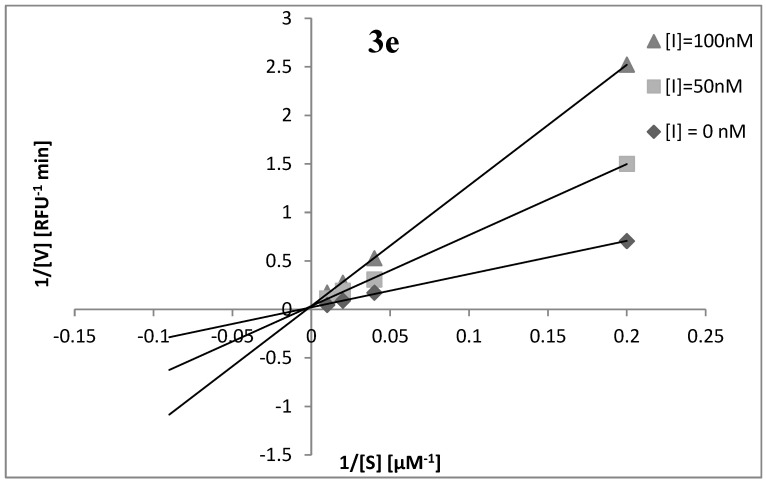
Lineweaver-Burk plots for inhibition of human neutrophil elastase in the presence of compound **3e**.

The first model is one in which the inhibitor binds better to the free enzyme (competitive mechanism), while the second model is one in which the inhibitor binds better to the enzyme-substrate complex (non-competitive mechanism). Next the secondary plots of slope versus concentration of test compounds were plotted to determine the enzyme-inhibitor dissociation constants K_i_, and similarly as was used to determine enzyme-substrate-inhibitor dissociation constants K_is_, the secondary plots of intercept versus concentration of tested compounds were plotted. The K_i_ values for the compounds **3c**, **3e**, **3f**, **3g**, and **3h** were lower than the K_is_ values, suggesting that these inhibitors bind more strongly to the free enzyme than the enzyme-substrate complex (mixed type I inhibition). For the remaining compounds, the K_i_ values are higher than the K_is_ values, indicating that these compounds have a higher affinity for the enzyme-substrate complex (mixed type II inhibition). The plot for the most active inhibitor **3e** is presented in Figure 2.

### 2.3. Molecular Docking Study

According to reference [32], the studied compounds are likely to be bound to protein by the formation of a covalent bond between serine and the azetidine-2,4-dione moiety, followed by the decomposition of the ligand molecule into two fragments. Thus, either standard (non-covalent) docking or the covalent docking involving the whole ligand molecule do not seem to be suitable for predicting the inhibition properties due to the following facts: (i) the low-energy ligand poses do not necessarily correspond to the conformations that facilitate the reaction with serine, thus, they may be chemically meaningless; (ii) after the ligand decomposition, the covalently bound ligand fragment is exactly the same for all considered compounds, thus, performing covalent docking would not produce any distinguishable results. To overcome these difficulties, we decided to carry out the standard docking that takes into account the complete molecular structure of all ligands but analyze only those poses that fulfil additional structural criteria. Namely, it was assumed that in order to enable the chemical reaction between the azetidine-2,4-dione moiety and serine (according to the structural data in the PDB database, covalent ligand bonding occurs always with contribution of Ser195) the distance between the serine hydroxyl oxygen atom and any of the two oxygens in the azetidine-2,4-dione group must be smaller than 0.3 nm. The poses that do not fulfil these cutoff-based criteria were discarded in further analysis. (Docking for sivelestat was carried out as well and an analogous distance-based cutoff was applied. According to reference [39], covalent binding occurs involving the carbonyl group; this was accounted for when defining the corresponding cutoff).

**Figure 3:**
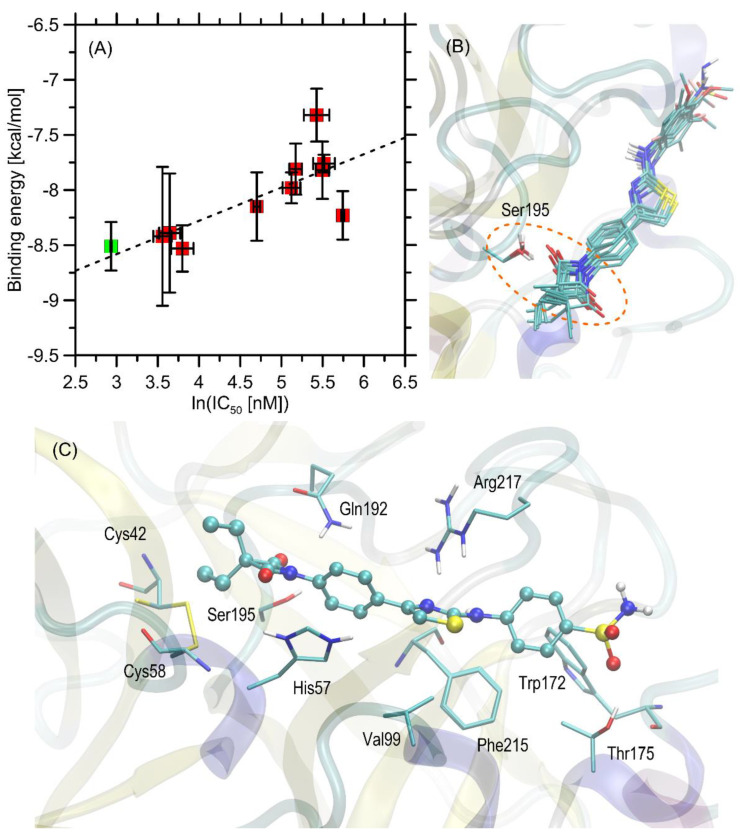
(**A**) The correlation between the calculated binding energies, averaged over the five elastase structures available in the PDB database, and the corresponding (experimental) values of IC_50_. The horizontal bars represent the standard deviation values within the set of binding energies. Green symbol corresponds to sivelestat whereas red ones correspond to the remaining compounds. (**B**) The superposed positions of all studied ligands (stick representation) in the binding cavity of the 1bma structure of elastase. All of these positions were identified as the optimal ones during the standard (non-covalent) docking procedure with additional conditions imposing the maximal distance of oxygen atoms at the azetidine-2,4-dione group and Ser195 as equal to 0.3 nm. (**C**) The location of the **3e** ligand molecule bound to the 1bma structure of elastase and found in the docking procedure. The ligand molecule is shown in a ball-and-stick representation, whereas all the closest amino-acid residues are represented by sticks. The description of the interaction types is given in the text.

This procedure can be considered as closely related to the covalent docking of whole ligand molecules or to docking with energy constraints on the interatomic distance. Here, the role of either additional covalent bonds or the constraint-inherent energy contribution is played by ‘logical constraint’, i.e., filtering out the ligand poses that fall outside the cutoff criteria. The molecular docking study was used to analyze the influence of the pharmacophore group on the activity of the studied compounds. The binding energies found during docking simulations are given in Table 2 and graphically illustrated in Figure 3. Each of the final energy values was averaged over the set of poses exhibiting the same structural orientation in the binding cavity, as confirmed by separate RMSD calculations and visual inspection (Figure 3B). All of the obtained ligand-protein interaction energies display similar magnitude, varying in the range of ~−7.3–−8.5 kcal/mol. The most favorable interactions are exhibited by compounds **3c**, **3e**, **3h** and sivelestat. These compounds displayed their high inhibition potencies in the experimental studies. Moreover, the binding energies calculated for all sets of compounds are fairly well correlated with the experimentally determined values of IC_50_ (Figure 3A). Thus, one can conclude that a reasonable agreement between the theoretical and experimental results exists, especially when considering the mechanism of ligand binding which can be tackled by a docking study only in an indirect manner. At the same time one can observe that the compounds exhibiting the highest IC_50_ values were not always correctly identified in the calculations; this is discussed later.

The relatively good correlation of the binding energies with the IC_50_ values suggests that the inhibition properties are largely dependent on the stage of ligand alignment in the binding cavity. In other words, the inhibition properties are correlated with the energy level of substrates of the reaction of ligand with Ser195.

The very small scatter of ligand-protein interaction energies across the studied set of compounds (1.2 kcal/mol) can be explained in terms of their high structural similarity (they differ only by the type of one substituent) combined with extremely close locations and orientations exhibited by them when interacting with the binding cavity.

The results of the docking studies were also analyzed with respect to the mechanistic protein-ligand interaction pattern that is essential for interpretation of the obtained binding energy values. The summary given below relies on analysing the ligand-protein contacts that take place if the distance between any corresponding atom pair is smaller than the arbitrarily accepted value of 0.4 nm. The provided description can be related to all the studied compounds due to their nearly identical orientations in the binding cavity, which was partially imposed by cutoff criteria (Figure 3). The accuracy limits in this context result mainly from the molecular topology of the ligand, i.e., different chemical character of a single moiety). The graphical illustration of the docking results (on example of **3e**) is given in Figure 3C.

The network of interactions responsible for distinguishing between potencies of particular compounds is created by Trp172, Thr175, Phe215 and (partially) Arg217. Independently of the compound, its limiting, aromatic substituent always maintains contact with Phe215 and Trp172 via attractive π-π or CH-π interactions. The presence of interactions with Thr175 is dependent on the character of the substituent and concerns only those compounds which contain polar groups in this region of molecule. Interactions with Arg217 occurs mainly with thiazole ring (as in Figure 3C and according to description below), however, the Arg217 sidechain is the most flexible among all sidechains considered explicitly during docking and can exhibit interactions also with the limiting substituent when the latter is polar. Although corresponding docking poses are only of the secondary type, the difference in energy levels is rather small (<0.3 kcal/mol), which suggests the consideration of this contact in the discussion. 

As the physical-chemical character of interactions formed by this crucial substituent and neighboring residues is not uniform and involves e.g., hydrogen bonding donation and acceptance, CH-π and π-π stacking, the detailed interpretation of the dependence of the moiety type on the compound potency is not straightforward. It seems that binding energies determined in the study result from the detailed balance of either favorable or non-favorable interactions of competitive character. 

For instance, the compounds exhibiting the most favorable binding energies (**3c**, **3e** and **3h**) have a distinctly different character of their substituents and the corresponding favorable interactions with protein can be rationalized differently. Compounds **3c** and **3e** contain a relatively large number of hydrogen bonding acceptors and/or donors which enables attractive interactions with Arg217 and Thr175. Compound **3h** does not contain any polar group in the corresponding substituent, but this is compensated by the enhanced magnitude of the π-π interactions of the condensed aromatic ring with Trp172 and Phe215. Compound **3g**, also bearing a larger, aromatic (indazole) ring is not capable of creating equally attractive interactions due to different substitution positions and, subsequently, the altered relative orientation of the ring with respect to the Phe215 sidechain. Finally, the energy balance, in particular the intermolecular contacts, depends partially on the factors that cannot be explicitly considered during docking e.g., the presence of solvent or the mobility of the protein backbone. The latter factor is demonstrated by a high variability of the determined binding energies and their dependence on the geometry of the protein backbone around Val99, as observed for compounds **3c** and **3e**.

The remaining ligand-protein interactions concern the polar interactions of Arg217 with thiazole ring (hydrogen bonding) and CH-π stacking also involving the thiazole ring and Val99 sidechain. The phenyl ring of the ligand maintains contact with His57 (aromatic π-π interactions) and Gln192. The same Gln192 is located in the vicinity of the azetidine-2,4-dione group where it is able to form hydrogen bonding with the nitrogen atom, stabilizing the arrangement of the azetidine-2,4-dione moiety with respect to Ser195. Thus, this type of interaction is probably essential in the context of the formation of covalent bonding with Ser195.

The part of the ligand molecule that contains the azetidine-2,4-dione moiety is especially crucial for the occurrence of the reaction with Ser195. Due to imposed logical constraints, the attractive, hydrogen bonding-mediated interactions are always observed in the case of one of the oxygen atoms in the azetidine-2,4-dione group and neighboring Ser195 sidechain. Apart from this contact (and the presence of Gln192, mentioned above) no other specific, attractive interactions can be ascribed to this part of the ligand molecule. The vicinity of the two cysteines (Cys42 and Cys58) and the aliphatic substituents of the azetidine-2,4-dione group seems to be only an opportunistic consequence of other protein-ligand interactions discussed above.

Thus, to summarize, the docking results showed that the potency of compounds is correlated with the energy level of the ligand-protein complex (substrates for the subsequent ligand-protein reactions resulting in the formation of a covalent bond) but only when a certain arrangement of the ligand is achieved, enabling for occurring further stages of process. This arrangement relies on close contact of the azetidine-2,4-dione group and the Ser195 sidechain, maintained through hydrogen bonding and stabilized by interactions with Gln192. The order of potencies of compounds considered in the present study depends on the interactions with Trp172, Thr175, Phe215 and (partially) Arg217.

### 2.4. Antiproliferative Activity

3,3-Diethylazetidine-2,4-dione based thiazoles **3a**–**3j** were assessed for their antiproliferative activity against a panel of four cancer cell lines: human biphenotypic B myelomonocytic leukemia (MV4-11), lung carcinoma (A549), human breast adenocarcinoma (MDA-MB-231), urinary bladder carcinoma (UMUC-3), and normal mouse fibroblast (BALB/3T3) cells. Biological studies were carried out using the 3-(4,5-dimethylthiazol-2-yl)-2,5-diphenyltetrazolium bromide (MTT) or sulforhodamine B (SRB) assays. The results are summarized in Table 3. Curves for the determination of IC_50_ values of antiproliferative activity of compounds **3a**–**3j** can be found in Supporting Information (see Figures S12–S16). All derivatives **3a**–**3j** showed high activity against MV-4-11 cells, with IC_50_ values between 4.59–7.15 µM. The antiproliferative activity of the tested compounds against human lung carcinoma (A549) cells is also very high, with IC_50_ values in the range of 5.59–7.31 µM. Some decrease in activity for this type of cancer was observed for compounds **3d** and **3h**, with IC_50_ values 15.28 and 9.20 µM, respectively. Our research also showed that compounds **3a**–**3c**, **3e** and **3g** have very high activity against human breast adenocarcinoma (MDA-MB-231) with IC_50_ values between 6.19 and 9.86 µM. Compounds **3f**, **3h**, **3i** and **3j** showed good activity with IC_50_ values in the range of 11.65–14.64 µM. In this type of cancer, compound **3d** showed the lowest activity with an IC_50_ value of 72.09 µM. Studies with urinary bladder carcinoma (UMUC-3) cells showed that four compounds **3b**, **3c**, **3e**, an **3g** showed good activity against this type of cancer, with IC_50_ values between 6.90 and 9.07 µM.

The less potent of the series for this cancer cell line was compounds **3a**, **3h** and **3i**, with IC_50_ values in the range of 10.67–13.31 µM. Compounds **3d**, **3f** and **3j** showed the lowest activity with IC_50_ value in the range of 19.70–62.73 µM. Due to the lack of a standard drug that is structurally similar to our compounds, we decided to use cisplatin, which is active against all cancer cell lines tested. Studies have shown that our compounds, except **3d**, are more active against MDA-MB-231 than cisplatin. Also, most compounds show less toxicity than cisplatin.

Anticancer drugs are toxic to cancer cells, but also show some toxicity to healthy cells. Therefore, in the next step, we determined the selectivity index (SI) of the cytotoxic activity of the 3,3-diethylazetidine-2,4-dione based thiazoles by comparing the cytotoxic activity (IC_50_) of compounds against the normal fibroblasts BALB/3T3 with the cytotoxic activity (IC_50_) of cancer cell lines (Table 4). The selectivity indexes for thiazoles **3a**–**3j** against MV4-11 and A-549 cells were in general much higher than for MDA-MB-231 and UMUC-3 cells. The compounds **3f**, **3h** and **3j** showed the higher selectivity index against all tested cancer cell lines.

### 2.5. Apoptosis and Cell Cycle Assays

**Figure 4:**
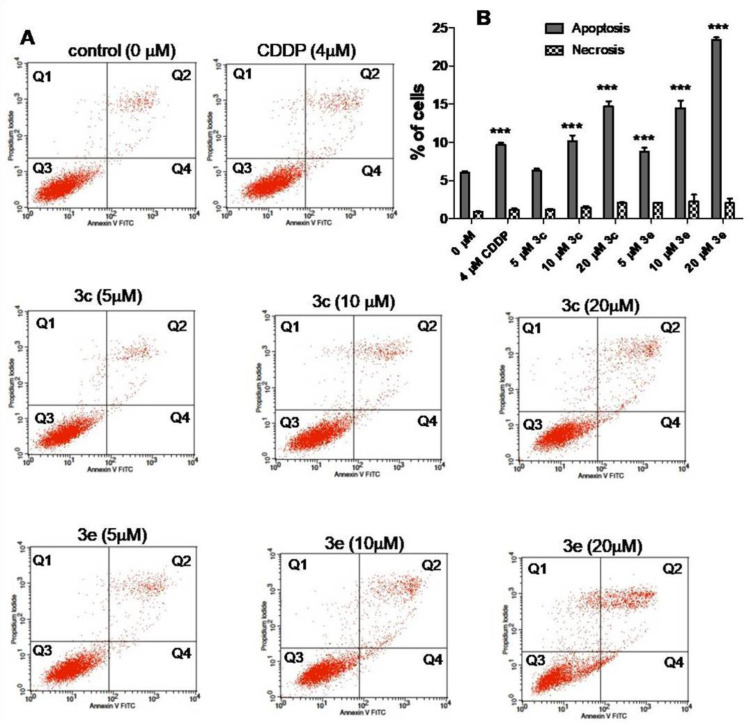
Effect of compounds **3c** and **3e** and cisplatin (CDDP) on apoptosis induction in the A549 cell line. After the 48-h exposure to the indicated concentrations of the compounds, the cells were stained with annexin (An) V-FITC)/propidium iodide (PI) and examined with flow cytometry. (**A**) The representative graphs obtained by flow cytometric analysis: dot plots indicate the percentage of An−/PI+ necrotic cells (Q1), An+ /PI+ late apoptotic cells (Q2), An−/PI− viable cells (Q3), and An+ /PI− early apoptotic cells (Q4). (**B**) Statistical analysis of the apoptotic (early + late apoptosis) and necrotic rate populations after the **3c**, **3e**, and cisplatin (CDDP) treatment. Data are presented as mean ± SD from three independent experiments; *** *p* < 0.001 in comparison to the control; one-way ANOVA followed by Dunnett’s post hoc test.

**Figure 5:**
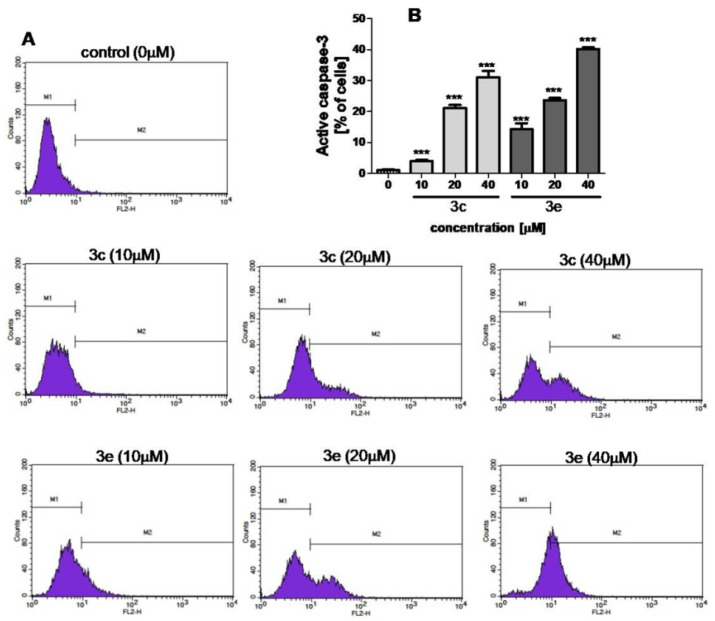
Flow cytometry analysis of active caspase-3 in A549 cells treated with compounds **3c** and **3e** for 48 h. (**A**) Representative histograms of A549 cell culture—M1 and M2 gates represent peaks for viable (caspase-3 negative cells) and apoptotic cell fractions (caspase-3 positive cells detected after cell staining with PE-conjugated anti-active caspase-3 antibodies), respectively. (**B**) Quantification of caspase-3 activity in A549 cell culture. The results are mean values ± SD of three independent experiments; statistical significance at *p* < 0.001 *** in comparison to the control; one-way ANOVA followed by Dunnett’s post hoc test.

**Figure 6:**
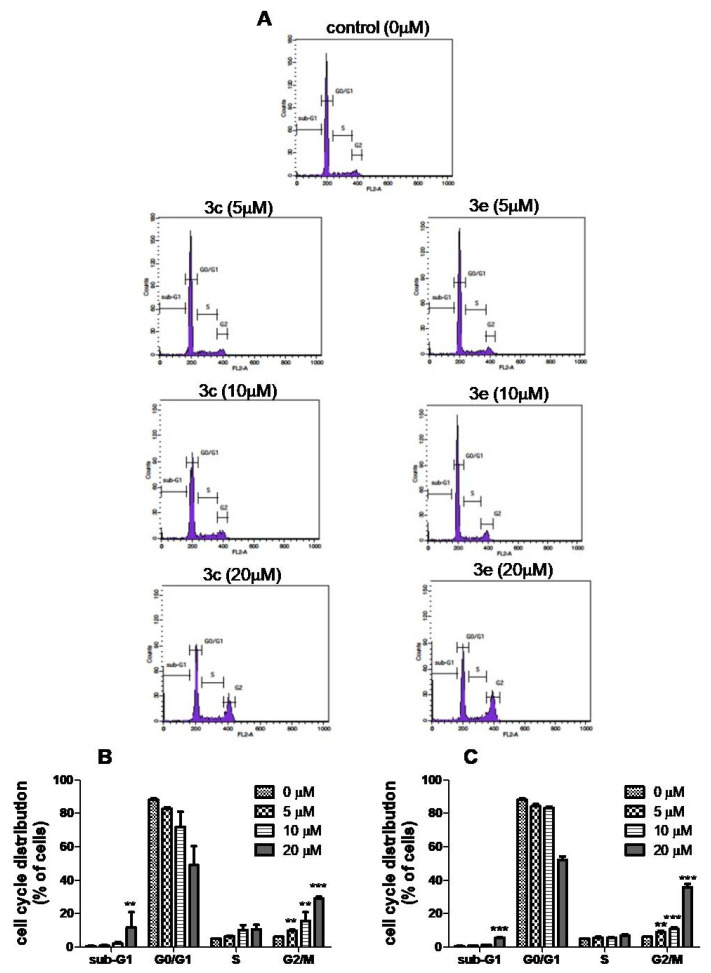
Effect of compounds **3c** and **3e** on cell cycle distribution in A549 cells. After the treatment with the indicated concentrations of compounds **3c** and **3e** for 48 h, the cells were stained with propidium iodide and analyzed by flow cytometry. (**A**) Representative DNA histograms of A549 cells treated with compounds **3c** and **3e**. (**B**,**C**) Statistical analysis of the percentages of A549 cells in the sub-G1, G0/G1, S, and G2/M phases after treatment with compounds **3c** and **3e**, respectively. Data are expressed as means ± SD from three independent experiments. ** *p* < 0.01 and *** *p* < 0.001 in comparison to the control; one-way ANOVA followed by Tukey’s post hoc test.

To explore the antiproliferative activity of the tested compounds, the influence of the most active ones, i.e., **3c** and **3e**, on apoptosis induction and caspase-3 activation and the distribution of cell cycle phases in the A549 cells after 48-h incubation were analyzed by flow cytometry. The rate of cell apoptosis and necrosis was detected using Annexin V-FITC/PI double staining. As shown in Figure 4A,B, both compounds were found to induce apoptosis in the A549 cells in a concentration-dependent manner, whereas no induction of necrosis was observed. Compound **3e** turned out to be a stronger inducer of apoptosis than **3c**. The percentage of A549 cells undergoing apoptosis increased significantly from 6.05 ± 0.17% in the control to 8.79 ± 0.50%, 14.45 ± 1.02%, and 23.39 ± 0.31% after the incubation with 5, 10, or 20 µM of the **3e**, respectively (Figure 4B). In turn, compound **3c** induced apoptosis only at the concentrations of 10 and 20 µM, and the percentage of cells undergoing apoptosis increased from 6.05 ± 0.17% in the control to 10.14 ± 0.76% and 14.67 ± 0.68%, respectively (Figure 4B). Moreover, compound **3e** at a concentration of 5 µM induced apoptosis to a similar level as cisplatin (CDDP) used at a concentration of 4 µM. To identify the mechanism of the **3c**- and **3e**-induced apoptosis in the A549 cells, the activation of effector caspase-3 was evaluated by flow cytometry. As shown in Figure 5A,B, the treatment of the cells with both compounds resulted in a concentration-dependent increase in the number of cells with active caspase 3. To further explore the antiproliferative activity of compounds **3c** and **3e**, the influence of both compounds on the distribution of cell cycle phases in the A549 cells was analyzed. The **3c** or **3e** treatment resulted in an increased number of A549 cells in the G2/M phase and the sub-G1 apoptotic subpopulation with a concomitant reduction of the cell number in the G0/G1 phase (Figure 6A–C). Compared to the control, compound **3c** used at a concentration of 5, 10, or 20 µM significantly elevated the G2/M fraction from 6.20 ± 0.23% to 9.69 ± 0.77%, 15.68 ± 5.31%, and 29.04 ± 1.09%, respectively (Figure 6B). The exposure of the cells to 0, 5, 10, and 20 µM of compound **3e** resulted in the accumulation of cells in the G2/M phase from 6.20 ± 0.23% to 9.08 ± 0.69%, 10.83 ± 1.06%, and 35.78 ± 0.46%, respectively (Figure 6C). The G2 checkpoint prevents cells from entering mitosis when DNA is damaged, providing an opportunity for repair and stopping the proliferation of damaged cells [41]. The cell cycle arrest at the G2/M phase indicates that the damage of intracellular DNA is difficult to repair [42]. Apoptosis is a crucial process involved in the regulation of tumor formation and in the treatment response; therefore, cancer therapy has been linked to activation of the apoptosis signal transduction pathway. Caspase-3, an executioner caspase, plays an important role in apoptosis and becomes a primary target for cancer treatment [43]. In conclusion, these results indicate that compounds **3c** and **3e** can induce cell cycle arrest at the G2/M phase and apoptosis via caspase-3 activation, leading to the inhibition of A549 cell proliferation.

### 2.6. Compounds Stability

**Figure 7:**
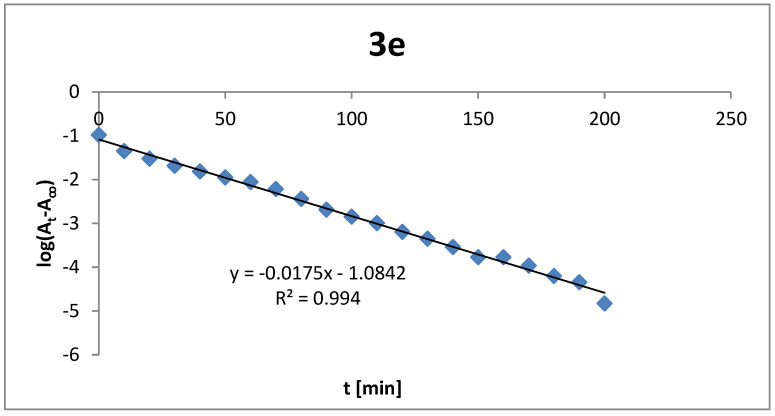
Semilogarithmic plots used to determine rate constants for spontaneous hydrolysis of compound **3e**.

**Figure 8:**
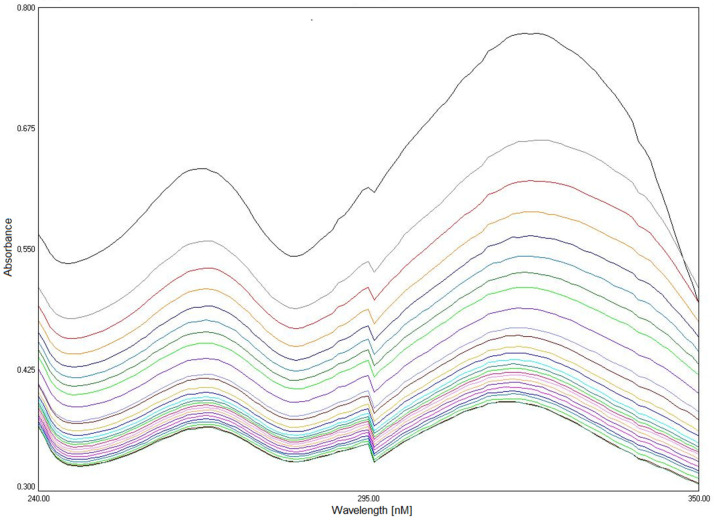
The changes in absorbance spectra of compound **3e** during spontaneous hydrolysis. The spectrum was monitored for 250 min with 10 min intervals in 0.05 M phosphate buffer (pH 7.3, 25 °C).

Testing the stability of new drugs is of great importance for obtaining a good therapeutic profile as well as the safety of their future use. Degradation impurities can reduce drug efficacy and generate undesirable side effects. Therefore, two of the most active 3,3-diethylazetidine-2,4-dione based thiazoles **3c** and **3e** were evaluated for chemical stability in an aqueous phosphate buffer at pH 7.3 imitating physiological conditions using spectrophotometric analysis (Table 5). An exemplary spectrum for compound **3e** is presented in Figure 7 and Figure 8. The absorbance maxima of compound **3c** at 293 nm and at 262 and 322 nm for compound **3e** decreased over time, indicating spontaneous hydrolysis with a t_1/2_ of 46.2 and 39.6 min, respectively. The tested compounds demonstrate high stability under physiological pH conditions.

## 3. Materials and Methods

### 3.1. Chemistry

All reactions were performed under a nitrogen atmosphere. All reagents and starting materials were purchased from commercial suppliers and used without further purification. The dichloromethane was dried over calcium hydride. ^1^H NMR (400 MHz) and ^13^C NMR (100 MHz) spectra were recorded on a Bruker Avance III multinuclear instrument. High resolution mass spectrometry measurements were performed using a Synapt G2-Si mass spectrometer (Waters) equipped with a quadrupole-Time-of-flight mass analyser. The mass spectrometer was operated in the positive ion detection mode. The results of the measurements were processed using the MassLynx 4.1 software (Waters) incorporated with the instrument. Melting points were determined in open glass capillaries and are uncorrected. Analytical TLC was performed using Macherey-Nagel Polygram Sil G/UV_254_ 0.2 mm plates. 2,2-Diethyl malonyl dichloride and appropriate thioureas were commercial materials (Merck).

#### 3.1.1. 1-(4-(2-Chloroacetyl)phenyl)-3,3-diethylazetidine-2,4-dione (**2**)

2,2-Diethyl malonyl dichloride (1.51 g, 1.32 mL, 7.67 mmol) was added to a stirred solution of 1-(4-aminophenyl)-2-chloroethanone (1) (1.0 g, 5.90 mmol) in dry dichloromethane (25 mL) and then triethylamine (1.55 g, 2.13 mL, 15.34 mmol) was added. The reaction mixture was stirred at room temperature for 20 h. The solvent was removed under reduced pressure, tetrahydrofuran (20 mL) was added and next the triethylamine hydrochloride was filtered off and the solvent was removed under reduced pressure. The product was purified on silica gel column chromatography (230–400 mesh) using (dichloromethane, R_f_ = 0.58) to reach the desired product: 1.30 g, 76%; mp 76–78 °C. ^1^H NMR (DMSO-*d*_6_, 400 MHz), δ (ppm): 0.98 (t, 6H, 2CH_3_, *J* = 7.7 Hz); 1.86 (q, 4H, 2CH_2_, *J* = 7.0 Hz); 5.19 (s, 2H, CH_2_); 7.88 (d, 2H, 2CH, *J* = 8.4 Hz); 8.12 (d, 2H, 2CH, *J* = 7.7 Hz). ^13^C NMR (DMSO-*d*_6_, 100 MHz), δ (ppm): 9.32 (2CH_3_); 23.18 (2CH_2_); 48.02 (C); 71.98 (C); 119.37 (2C); 130.55 (2C); 132.64 (C); 137.54 (C); 171.99 (2C); 190.96 (C). 

#### 3.1.2. 1-(4-(2-(4-Chlorophenylamino)thiazol-4-yl)phenyl)-3,3-diethylazetidine-2,4-dione (**3a**) Typical Procedure

*N*-(4-Chlorophenyl)thiourea (0.187 g, 1.0 mmol) was added to a stirred solution of 1-(4-(2-chloroacetyl)phenyl)-3,3-diethylazetidine-2,4-dione (**2**) (0.294 g, 1.0 mmol) in absolute ethyl alcohol (20 mL). The reaction mixture was stirred under reflux for 20 h. Next, the reaction mixture was added to water (50 mL) and neutralized with NaHCO_3_ solution. The separate precipitate was collected by filtration and dried in vacuum to afford the desired product: 0.35 g, 82%; mp 164–166 °C, eluent: (dichloromethane/methanol (95:5); R_f_ = 0.81). ^1^H NMR (DMSO-*d*_6_, 400 MHz), δ (ppm): 0.99 (t, 6H, 2CH_3_, *J* = 7.7 Hz); 1.85 (q, 4H, 2CH_2_, *J* = 7.7 Hz); 7.39 (d, 2H, 2CH_,_ *J* = 9.1 Hz); 7.42 (s, 1H, CH); 7.76–7.79 (m, 4H, 4CH); 8.05 (d, 2H, 2CH_,_ *J* = 8.4 Hz); 10.47 (s, 1H, NH). ^13^C NMR (DMSO-*d*_6_, 100 MHz), δ (ppm): 9.39 (2CH_3_); 23.31 (2CH_2_); 71.64 (C); 104.62 (C); 118.86 (2C); 119.91 (2C); 125.06 (C); 127.17 (2C); 129.30 (2C); 132.63 (C); 133.44 (C); 140.46 (C); 149.53 (C); 163.36 (C); 172.08 (2C). ESI-HRMS (*m/z*) calculated for C_22_H_21_ClN_3_O_2_S: 426.1043 [M + H]^+^. Found: 426.1039 [M + H]^+^.

#### 3.1.3. 3,3-Diethyl-1-(4-(2-(4-fluorophenylamino)thiazol-4-yl)phenyl)azetidine-2,4-dione (**3b**) 

Yield: 0.14 g, 68%, (dichloromethane/methanol (95:5), R_f_ = 0.81); mp 155–157 °C. ^1^H NMR (DMSO-*d*_6_, 400 MHz), δ (ppm): 0.97 (t, 6H, 2CH_3_, *J* = 8.0 Hz); 1.83 (q, 4H, 2CH_2_, *J* = 7.6 Hz); 7.17 (t, 2H, 2CH_,_ *J* = 8.8 Hz); 7.37 (s, 1H, CH); 7.18 (q, 2H, 2CH, *J* = 4.8 Hz); 7.75 (d, 2H, 2CH_,_ *J* = 9.2 Hz); 8.02 (d, 2H, 2CH, *J* = 9.6 Hz); 10.31 (s, 1H, NH). ^13^C NMR (DMSO-*d*_6_, 100 MHz), δ (ppm): 9.40 (2CH_3_); 23.30 (2CH_2_); 71.64 (C); 104.20 (C); 115.99 (d, 2C, *J* = 20.9 Hz); 118.95 (d, 2C, *J* = 9.4 Hz); 119.93 (2C); 127.14 (2C); 132.49 (C); 133.53 (C); 138.13 (C); 149.45 (C); 157.37 (d, C, *J* = 227.8 Hz); 163.88 (C); 172.21 (2C). ESI-HRMS (*m/z*) calculated for C_22_H_21_FN_3_O_2_S: 410.1339 [M + H]^+^. Found: 410.1335 [M + H]^+^.

#### 3.1.4. 3,3-Diethyl-1-(4-(2-(3,4,5-trimethoxyphenylamino)thiazol-4-yl)phenyl)azetidine-2,4-dione (**3c**) 

Yield: 0.34 g, 71%, (dichloromethane/methanol (95:5), R_f_ = 0.61); mp 188–190 °C. ^1^H NMR (DMSO-*d*_6_, 400 MHz), δ (ppm): 0.97 (t, 6H, 2CH_3_, *J* = 7.6 Hz); 1.83 (q, 4H, 2CH_2_, *J* = 6.8 Hz); 3.61 (s, 3H, CH_3_); 3.80 (s, 6H, 2CH_3_); 7.11 (s, 2H, 2CH); 7.37 (s, 1H, CH); 7.75 (d, 2H, 2CH_,_ *J* = 8.4 Hz); 8.01 (d, 2H, 2CH, *J* = 8.8 Hz); 10.24 (s, 1H, NH). ^13^C NMR (DMSO-*d*_6_, 100 MHz), δ (ppm): 9.40 (2CH_3_); 23.34 (2CH_2_); 56.10 (2CH_3_); 60.61 (CH_3_); 71.68 (C); 95.19 (2C); 104.07 (C); 120.03 (2C); 126.95 (2C); 132.43 (C); 132.49 (C); 133.59 (C); 137.75 (C); 149.35 (C); 153.48 (2C); 163.37 (C); 172.16 (2C). ESI-HRMS (*m/z*) calculated for C_25_H_28_N_3_O_5_S: 482.1750 [M + H]^+^. Found: 482.1749 [M + H]^+^.

#### 3.1.5. 3,3-Diethyl-1-(4-(2-(perfluorophenylamino)thiazol-4-yl)phenyl)azetidine-2,4-dione (**3d**) 

Yield: 0.43 g, 89%, (dichloromethane/methanol (95:5), R_f_ = 0.80); mp 162–164 °C. ^1^H NMR (DMSO-*d*_6_, 400 MHz), δ (ppm): 0.96 (t, 6H, 2CH_3_, *J* = 7.6 Hz); 1.82 (q, 4H, 2CH_2_, *J* = 7.6 Hz); 7.42 (s, 1H, CH); 7.70 (d, 2H, 2CH_,_ *J* = 8.4 Hz); 7.87 (d, 2H, 2CH, *J* = 8.8 Hz); 10.10 (s, 1H, NH). ^13^C NMR (DMSO-*d*_6_, 100 MHz), δ (ppm): 9.35 (2CH_3_); 23.29 (2CH_2_); 71.62 (C); 106.24 (C); 119.87 (2C); 120.80 (C); 126.34 (C); 127.10 (2C); 132.62 (C); 133.14 (C); 141.44 (2C); 143.86 (2C); 149.13 (C); 164.64 (C); 172.10 (2C). ESI-HRMS (*m/z*) calculated for C_22_H_17_F_5_N_3_O_2_S: 482.0962 [M + H]^+^. Found: 482.0963 [M + H]^+^.

#### 3.1.6. 4-(4-(4-(3,3-Diethyl-2,4-dioxoazetidin-1-yl)phenyl)thiazol-2-ylamino)benzenesulfo-namide (**3e**) 

Yield: 0.36 g, 82%, (dichloromethane/methanol (95:5), R_f_ = 0.39); mp 130–132 °C. ^1^H NMR (DMSO-*d*_6_, 400 MHz), δ (ppm): 1.00 (t, 6H, 2CH_3_, *J* = 7.7 Hz); 1.86 (q, 4H, 2CH_2_, *J* = 7.7 Hz); 7.22 (s, 2H, NH_2_); 7.51 (s, 1H, CH); 7.80 (d, 2H, 2CH_,_ *J* = 6.3 Hz); 7.82 (d, 2H, 2CH, *J* = 6.3 Hz); 7.88 (d, 2H, 2CH_,_ *J* = 9.1 Hz); 8.09 (d, 2H, 2CH, *J* = 8.4 Hz); 10.74 (s, 1H, NH). ^13^C NMR (DMSO-*d*_6_, 100 MHz), δ (ppm): 9.40 (2CH_3_); 23.31 (2CH_2_); 71.66 (C); 105.37 (C); 116.68 (2C); 119.97 (2C); 127.26 (2C); 127.65 (2C); 132.67 (C); 133.34 (C); 136.54 (C); 144.22 (C); 149.66 (C); 163.00 (C); 172.16 (2C). ESI-HRMS (*m/z*) calculated for C_22_H_23_N_4_O_4_S_2_: 471.1161 [M + H]^+^. Found: 471.1157 [M + H]^+^.

#### 3.1.7. 1-(4-(2-(3,5-Dimethylphenylamino)thiazol-4-yl)phenyl)-3,3-diethylazetidine-2,4-dione (**3f**) 

Yield: 0.20 g, 99%, (dichloromethane/methanol (95:5), R_f_ = 0.87); mp 140–143 °C. ^1^H NMR (DMSO-*d*_6_, 400 MHz), δ (ppm): 0.97 (t, 6H, 2CH_3_, *J* = 8.0 Hz); 1.83 (q, 4H, 2CH_2_, *J* = 8.0 Hz); 2.62 (s, 6H, 2CH_3_); 6.61 (s, 1H, CH); 7.29 (s, 2H, 2CH); 7.35 (s, 1H, CH); 7.76 (d, 2H, 2CH_,_ *J* = 9.2 Hz); 8.01 (d, 2H, 2CH, *J* = 9.2 Hz); 10.15 (s, 1H, NH). ^13^C NMR (DMSO-*d*_6_, 100 MHz), δ (ppm): 9.41 (2CH_3_); 21.75 (2CH_3_); 23.33 (2CH_2_); 71.68 (C); 104.02 (C); 115.30 (2C); 120.00 (2C); 123.56 (C); 127.08 (2C); 132.49 (C); 133.58 (C); 138.48 (2C); 141.48 (C); 149.36 (C); 163.93 (C); 172.19 (2C). ESI-HRMS (*m/z*) calculated for C_24_H_26_N_3_O_2_S: 420.1746 [M + H]^+^. Found: 420.1747 [M + H]^+^.

#### 3.1.8. 1-(4-(2-(1*H*-Indazol-5-ylamino)thiazol-4-yl)phenyl)-3,3-diethylazetidine-2,4-dione (**3g**) 

Yield: 0.16 g, 83%, (dichloromethane/methanol (95:5), R_f_ = 0.42); mp 186–188 °C. ^1^H NMR (DMSO-*d*_6_, 400 MHz), δ (ppm): 0.98 (t, 6H, 2CH_3_, *J* = 7.2 Hz); 1.84 (q, 4H, 2CH_2_, *J* = 7.6 Hz); 7.33 (s, 1H, CH); 7.39–7.43 (m, 1H, CH); 7.48–7.53 (m, 1H, CH); 7.77 (d, 2H, 2CH_,_ *J* = 8.8 Hz); 8.05–8.07 (m, 3H, 3CH); 8.30 (d, 1H, CH, *J* = 2.0 Hz); 10.23 (s, 1H, NH); 12.94 (s, 1H, NH). ^13^C NMR (DMSO-*d*_6_, 100 MHz), δ (ppm): 9.43 (2CH_3_); 23.32 (2CH_2_); 71.67 (C); 103.56 (C); 107.14 (C); 111.10 (C); 119.87 (C); 119.94 (2C); 123.55 (C); 127.17 (2C); 132.46 (C); 133.67 (C); 133.74 (C); 135.08 (C); 136.75 (C); 149.53 (C); 164.58 (C); 172.16 (2C). ESI-HRMS (*m/z*) calculated for C_23_H_22_N_5_O_2_S: 432.1494 [M + H]^+^. Found: 432.1491 [M + H]^+^.

#### 3.1.9. 3,3-Diethyl-1-(4-(2-(naphthalen-1-ylamino)thiazol-4-yl)phenyl)azetidine-2,4-dione (**3h**) 

Yield: 0.14 g, 63%, (dichloromethane/methanol (95:5), R_f_ = 0.89); mp 69–71 °C. ^1^H NMR (DMSO-*d*_6_, 400 MHz), δ (ppm): 0.98 (t, 6H, 2CH_3_, *J* = 7.2 Hz); 1.83 (q, 4H, 2CH_2_, *J* = 7.6 Hz); 7.31 (s, 1H, CH); 7.50–7.58 (m, 3H, 3CH); 7.65–7.69 (m, 1H, CH); 7.75 (d, 2H, 2CH_,_ *J* = 7.6 Hz); 7.91–7.96 (m, 1H, CH); 8.01 (d, 2H, 2CH, *J* = 8.8 Hz); 8.25–8.33 (m, 2H, 2CH); 10.20 (s, 1H, NH). ^13^C NMR (DMSO-*d*_6_, 100 MHz), δ (ppm): 9.40 (2CH_3_); 23.31 (2CH_2_); 71.64 (C); 104.65 (C); 117.06 (C); 119.95 (2C); 122.62 (C); 123.85 (C); 126.17 (C); 126.45 (C); 126.59 (C); 126.64 (C); 127.12 (2C); 128.76 (C); 132.52 (C); 133.70 (C); 134.48 (C); 137.14 (C); 149.44 (C); 166.18 (C); 172.18 (2C). ESI-HRMS (*m/z*) calculated for C_26_H_24_N_3_O_2_S: 442.1589 [M + H]^+^. Found: 442.1581 [M + H]^+^.

#### 3.1.10. 3,3-Diethyl-1-(4-(2-(4-(trifluoromethyl)phenylamino)thiazol-4-yl)phenyl)-azetidine-2,4-dione (**3i**) 

Yield: 0.24 g, 52%, (dichloromethane/methanol (95:5), R_f_ = 0.92); mp 170–172 °C. ^1^H NMR (DMSO-*d*_6_, 400 MHz), δ (ppm): 0.99 (t, 6H, 2CH_3_, *J* = 7.0 Hz); 1.85 (q, 4H, 2CH_2_, *J* = 7.7 Hz); 7.50 (s, 1H, CH); 7.70 (d, 2H, 2CH_,_ *J* = 8.4 Hz); 7.79 (d, 2H, 2CH, *J* = 8.4 Hz); 7.94 (d, 2H, 2CH, *J* = 8.4 Hz); 8.08 (d, 2H, 2CH, *J* = 8.4 Hz); 10.76 (s, 1H, NH). ^13^C NMR (DMSO-*d*_6_, 100 MHz), δ (ppm): 9.37 (2CH_3_); 23.29 (2CH_2_); 71.66 (C); 105.37 (C); 117.05 (2C); 119.82 (2C); 123.78 (C); 126.46 (C); 126.80 (2C); 127.22 (2C); 132.72 (C); 133.32 (C); 144.79 (C); 149.62 (C); 162.96 (C); 172.15 (2C). ESI-HRMS (*m/z*) calculated for C_23_H_21_F_3_N_3_O_2_S: 460.1307 [M + H]^+^. Found: 460.1302 [M + H]^+^.

#### 3.1.11. 3,3-Diethyl-1-(4-(2-(2,4,6-trichlorophenylamino)thiazol-4-yl)phenyl)azetidine-2,4-dione (**3j**) 

Yield: 0.22 g, 89%, (dichloromethane/methanol (95:5), R_f_ = 0.91); mp 78–80 °C. ^1^H NMR (DMSO-*d*_6_, 400 MHz), δ (ppm): 0.96 (t, 6H, 2CH_3_, *J* = 7.2 Hz); 1.82 (q, 4H, 2CH_2_, *J* = 7.6 Hz); 7.30 (s, 1H, CH); 7.70 (d, 2H, 2CH_,_ *J* = 8.0 Hz); 7.82 (s, 2H, 2CH); 7.85 (d, 2H, 2CH, *J* = 8.4 Hz); 9.86 (s, 1H, NH). ^13^C NMR (DMSO-*d*_6_, 100 MHz), δ (ppm): 9.39 (2CH_3_); 23.28 (2CH_2_); 71.59 (C); 104.84 (C); 119.88 (2C); 127.07 (2C); 129.36 (4C); 132.28 (C); 132.53 (C); 133.40 (C); 134.71 (C); 149.46 (C); 166.16 (C); 172.12 (2C). ESI-HRMS (*m/z*) calculated for C_22_H_19_Cl_3_N_3_O_2_S: 494.0264 [M + H]^+^. Found: 494.0261 [M + H]^+^.

### 3.2. Human Neutrophil Elastase Inhibition Assay

#### Kinetic Analysis of the Inhibition of Human Neutrophil Elastase

Kinetic studies were conducted in the presence of different concentrations of substrate (5, 25, 50 and 100 µM) and test compounds (in the range of 0–500 nM). The reaction conditions were the same as in the HNE inhibition assay and the reaction was monitored for 10 min at 25 °C with the same E_x_ an E_m_ wavelengths. By Lineweaver–Burk plots, kinetic values such as the Michaelis–Menten constants and maximum velocity were determined. Next, two inhibition constants for inhibitor binding with free enzyme or enzyme–substrate complex (K_i_ and K_is_) were obtained by plotting the secondary plot of the slopes of the determined straight lines or vertical intercept (1/V_max_) versus inhibitor concentration [44]. 

### 3.3. Molecular Docking Study

The ligand molecules were prepared manually by using the Avogadro 1.1.1 software [45] and optimized within the UFF force field [46] (3000 steps, conjugate gradient algorithm). The flexible, optimized ligands were docked into the binding pockets of the high-resolution elastase structures found in the following five PDB entries: 1bma (X-ray resolution: 0.192 nm), 1hv7 (X-ray resolution: 0.17 nm), 1qnj (X-ray resolution: 0.11 nm), 2de9 (X-ray resolution: 0.13 nm) and 2h1u (X-ray resolution: 0.16 nm). The AutoDock Vina software [47] was applied in all docking simulations. The procedure of docking was carried out within the cuboid region which covered the whole co-crystallized ligand present in the 2h1u PDB structure as well as the closest amino-acid residues that exhibit contact with that ligand (of dimensions: 20 × 20 × 20 Å^3^). In order to provide a sufficient number of conformationally diverse ligand arrangements, the number of possible poses generated during a single run was increased to 15, whereas the energy threshold between the highest- and lowest-ranked poses was increased to 5 kcal/mol. Apart from that, all the default procedures and algorithms implemented in AutoDock Vina were applied during the docking procedure. The predicted binding energies were averaged over all protein structures applied for docking, whereas only the ligand poses exhibiting the same desired structural features regarding the distance with respect to Ser195 were subjected to calculations of such average energy. The more favorable binding mode is associated with the lower binding energy value; only the lowest energy values corresponding to the given ligand and poses fulfilling the cutoff criteria were accounted for during subsequent analysis. The visual inspection of the location and orientation of the docked ligands, in order to control the uniformity of the binding pattern, was performed. The docking methodology was initially validated by the docking simulation of the non-covalently-bound ligand molecule originally included in one of the studied protein structures (i.e. PDB:2h1u). Details of the procedure are given in our previous work [25].

### 3.4. Antiproliferative Activity

Cell preparation, an in vitro antiproliferative assay, and MTT and SRB cytotoxic tests were performed according to the literature [35,48,49,50].

### 3.5. Apoptosis and Cell Cycle Assays

The fluorescence-activated cell sorting (FACS) technique was employed to analyze the 3c- and 3e-induced cell death, the activation of executive caspase-3, and the cell cycle in the A549 cells. The quantitative analysis of apoptosis and necrosis was performed using an Annexin V-fluorescein isothiocyanate (FITC)/ propidium iodide (PI) apoptosis kit (BD Biosciences, BD Pharmingen™, San Jose, CA, USA) as previously described [36]. To determine the active form of caspase-3, the A549 cells were plated into 6-well plates at a density of 6 × 10^5^ cells/well. The next day, the growth medium was replaced with a fresh one supplemented with compound **3c** or **3e** (10, 20, and 40 µM) or the DMSO vehicle (0.1%; control cells). After 48-h incubation, the samples were harvested and the level of active caspase-3 was determined using the phycoerythrin (PE) Active Caspase-3 Apoptosis Kit (BD Pharmingen™) according to the manufacturer’s instructions. The stained cells were analyzed using FACS Calibur, and data were analyzed using Cell Quest Pro Version 6.0 (BD Biosciences, San Jose, CA, USA) for the Macintosh operating system. The results were calculated as a percent of cells with active caspase-3 among all the analyzed cells. The cell cycle analysis was performed by determination of the DNA contents on the basis of PI staining as previously described [36].

### 3.6. Analysis of Compounds Stability

Spontaneous hydrolysis of most active compounds was determined as previously described [51]. The kinetics hydrolysis of the compounds was evaluated at 25 °C in 0.05 M phosphate buffer, pH 7.3, by measuring changes in absorbance spectra during incubation using a T60U spectrophotometer (PG Instruments). The absorbance (A_t_) at characteristic absorption maxima for each compound was measured at 10 min intervals until there was no further decrease in absorbance (A_ꝏ_). Using these measurements, semilogarithmic plots of the dependence of log(A_t_–A_ꝏ_) on time were prepared, and k’ values were determined from the slope of these plots according to first order reaction kinetics. Half-conversion times were calculated using equation: t_1/2_ = 0.693/k’.

## 4. Conclusions

In the present work, we developed an efficient method for the synthesis of new thiazole derivatives containing 3,3-diethylazetidine-2,4-dione moiety. The resulting compounds are promising human neutrophil elastase inhibitors. The best results were obtained introducing the trimethoxyphenyl, benzenesulfonamide and naphthyl groups, which resulted in corresponding HNE inhibitors with IC_50_ values of 35.02–44.59 nM. Additionally, the tested compounds demonstrate high stability under physiological conditions. The molecular docking study showed good correlation of the binding energies with the IC_50_ values, which, as a consequence of accepted logical constraint, suggests that the potency of compounds is determined during ligand alignment in the binding cavity prior to its covalent binding via Ser195. Additionally, the obtained derivatives show a high and broad spectrum of antiproliferative activity, which suggests that these new drugs, viaHNE inhibition, will be active against the cancers that produce immunoreactive HNE. Additionally, compounds **3c** and **3e** can induce cell cycle arrest at the G2/M phase and apoptosis via caspase-3 activation, leading to the inhibition of A549 cell proliferation.

## Figures and Tables

**Table 1:** Human neutrophil elastase inhibitory activity of 3,3-diethylazetidine-2,4-dione based thiazoles **3a**–**3j** and kinetic analysis of their mechanism of action.

Thiazole Derivatives	IC_50_ ± SD [nM]	Dose [µM]	$V_{\max}$	K_m_	Inhibition Type	K_i_ [nM]	K_is_ [nM]
**3a**	227.29 ± 35.08	100.00 250.00	53.19 55.25	195.19 264.55	mixed	590.41	426.00
**3b**	176.28 ± 13.49	100.00 250.00	63.29 15.95	280.95 112.31	mixed	72.15	65.50
**3c**	38.25 ± 4.91	50.00 100.00	40.65 25.58	193.65 207.26	mixed	66.06	99.50
**3d**	110.02 ± 4.41	100.00 250.00	30.49 10.59	126.00 90.08	mixed	138.22	49.33
**3e**	35.02 ± 3.88	50.00 100.00	28.82 28.57	210.77 355.14	mixed	35.78	240.00
**3f**	243.96 ± 19.40	100.00 250.00	44.64 37.31	186.93 204.42	mixed	144.40	426.00
**3g**	312.19 ± 5.76	250.00 500.00	56.50 15.97	229.24 74.35	mixed	137.24	171.25
**3h**	44.59 ± 6.04	50.00 100.00	29.15 15.04	190.67 184.09	mixed	33.94	46.50
**3i**	167.57 ± 18.09	100.00 250.00	75.76 32.26	284.78 154.24	mixed	596.64	425.00
**3j**	248.74 ± 32.76	250.00 500.00	24.63 6.88	132.14 71.54	mixed	209.35	38.00
**Sivelestat**	18.78 ± 0.13	**─**	**─**	**─**	**─**	**─**	**─**

V_max_—maximum velocity, K_m_—inhibition constant, K_i_—enzyme-inhibitor dissociation constants, K_is_—enzyme-substrate-inhibitor dissociation constants.

**Table 2:** The docking results with respect to the studied ligands. The calculations were averaged over the five structures available in the PDB database; the corresponding standard deviations are given as well.

Compound	Binding Energy ± SD [kcal/mol]
**3a**	−7.32 ± 0.24
**3b**	−7.81 ± 0.23
**3c**	−8.39 ± 0.54
**3d**	−8.15 ± 0.31
**3e**	−8.42 ± 0.63
**3f**	−7.82 ± 0.26
**3g**	−8.23 ± 0.22
**3h**	−8.53 ± 0.21
**3i**	−7.98 ± 0.14
**3j**	−7.76 ± 0.08
**Sivelestat**	−8.51 ± 0.22

**Table 3:** Antiproliferative activity of thiazoles **3a**–**3j** against human biphenotypic B myelomonocytic leukemia (MV4-11), lung carcinoma (A549), human breast adenocarcinoma (MDA-MB-231), urinary bladder carcinoma (UMUC-3), and normal mouse fibroblast BALB/3T3 cells.

Thiazole Derivatives	IC_50_ ± SD [µM]				
	MV-4-11	A-549	MDA-MB-231	UMUC-3	Balb/3T3
**3a**	7.15 ± 0.94	7.01 ± 0.47	9.86 ± 0.94	10.68 ± 1.41	10.87 ± 1.18
**3b**	5.92 ± 1.71	7.31 ± 0.46	9.34 ± 1.12	9.07 ± 0.49	11.49 ± 1.47
**3c**	4.59 ± 2.03	6.40 ± 0.08	6.38 ± 0.71	6.90 ± 0.21	7.42 ± 1.04
**3d**	5.40 ± 2.06	15.28 ± 4.98	72.09 ± 25.98	62.73 ± 9.98	46.52 ± 4.99
**3e**	6.23 ± 0.42	6.32 ± 0.89	6.42 ± 1.70	7.49 ± 0.64	6.57 ± 0.21
**3f**	5.89 ± 1.67	7.28 ± 1.07	12.48 ± 1.79	19.70 ± 5.49	23.83 ± 4.53
**3g**	5.38 ± 0.46	5.59 ± 0.16	6.19 ± 0.42	7.89 ± 0.23	6.03 ± 0.46
**3h**	6.64 ± 1.59	9.20 ± 1.36	14.64 ± 3.17	13.31 ± 1.81	22.44 ± 8.62
**3i**	5.47 ± 1.52	6.42 ± 0.72	11.65 ± 3.70	10.67 ± 3.05	10.13 ± 0.87
**3j**	5.11 ± 1.42	6.21 ± 0.73	14.30 ± 3.45	25.29 ± 9.94	34.48 ± 22.72
**cisplatin**	1.33 ± 0.17	4.09 ± 1.00	19.43 ± 6.80	1.73 ± 0.17	8.04 ± 3.16

IC_50_—compound concentration leading to 50% inhibition of cell proliferation. IC values were calculated for each experiment separately (experiments were repeated 3–5 times) and data are presented as mean values ± SD, calculated using Prolab-3 system based on Cheburator 0.4 software [40].

**Table 4:** The selectivity index (SI) of tested compounds. The SI index = IC_50_ for normal cell line (Balb/3T3)/IC_50_ for appropriate cancer cell line.

Thiazole Derivatives	Cell Lines/Calculated Selectivity Index SI			
	MV-4-11	A-549	MDA-MB-231	UMUC-3
**3a**	1.52	1.55	1.10	1.02
**3b**	1.94	1.57	1.23	1.27
**3c**	1.62	1.16	1.16	1.07
**3d**	8.61	3.04	0.64	0.74
**3e**	1.05	1.04	1.02	0.88
**3f**	4.04	3.27	1.90	1.21
**3g**	1.12	1.08	0.97	0.76
**3h**	3.38	2.44	1.53	1.69
**3i**	1.85	1.58	0.87	0.95
**3j**	6.75	5.55	2.41	1.36

A beneficial SI > 1.0 indicates a drug with efficacy against cancer cells greater than toxicity against normal cells.

**Table 5:** Half-life (t1/2) for the spontaneous hydrolysis of selected compounds **3c** and **3e**.

Thiazole Derivatives	λ [nm]	k [min^−1^]	t_1/2_ [min]
**3c**	293	0.015	46.2
**3e**	262, 322	0.0175	39.6

## Data Availability

Not applicable.

## Supplementary Materials

The following supporting information can be downloaded at: https://www.mdpi.com/article/10.3390/ijms23147566/s1.
