# Supplementary material for: Discovery of New 3,3-Diethylazetidine-2,4-dione Based Thiazoles as Nanomolar Human Neutrophil Elastase Inhibitors with Broad-Spectrum Antiproliferative Activity
Source: Int J Mol Sci. 2022 Jul 8;23(14):7566. doi: 10.3390/ijms23147566 (PMC9321231; doi:10.3390/ijms23147566)
Supplement: Supplementary file 1 [file ijms-23-07566-s001.zip › ijms-1784669-supplementary.pdf]

Supporting Information

Discovery of new 3,3-diethylazetidine-2,4-dione based thiazoles as nanomolar human neutrophil elastase inhibitors with broad-spectrum antiproliferative activity

Beata Donarska [1], Marta Świtalska [2], Joanna Wietrzyk [2], Wojciech Płaziński [3,4], Magdalena Mizerska-Kowalska [5], Barbara Zdzisińska [5], Krzysztof Z. Łączkowski [1,*]

[1]*Department of Chemical Technology and Pharmaceuticals, Faculty of Pharmacy, Collegium Medicum, Nicolaus Copernicus University, Jurasza 2, 85-089 Bydgoszcz, Poland,*
[2]*Hirszfeld Institute of Immunology and Experimental Therapy, Polish Academy of Sciences, Rudolfa Weigla 12, 53-114 Wrocław, Poland,*
[3]*Jerzy Haber Institute of Catalysis and Surface Chemistry, Polish Academy of Sciences, Niezapominajek 8, 30-239, Cracow, Poland,*
[4]*Department of Biopharmacy, Medical University of Lublin, Chodzki 4a, 20-093 Lublin, Poland,*
[5]*Department of Virology and Immunology, Faculty of Biology and Biotechnology, Maria Curie-Skłodowska University, Akademicka 19 Street, 20-033 Lublin, Poland*

*Corresponding author: Krzysztof Z. Łączkowski, *e-mail*: krzysztof.laczkowski@cm.umk.pl

Contents

1. Curves for the determination of IC_{50} values for inhibition of HNE activity of compounds **3a**-**3j** and sivelestat (Figures S1–S11).

2. Curves for the determination of IC_{50} values of antiproliferative activity of compounds **3a**-**3j** (Figures S12–S16).

3. ^1H, ^{13}C NMR and HRMS spectra of compounds **2** and **3a**-**3j**.

Figure S1. Curves for the determination of IC_{50} values for inhibition of HNE activity of compound **3a**.

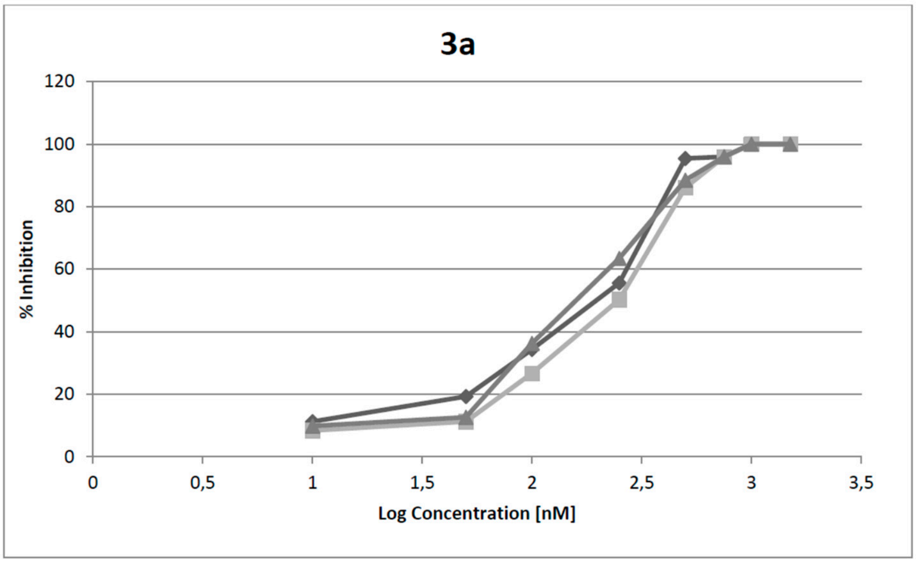

Figure S2. Curves for the determination of IC_{50} values for inhibition of HNE activity of compound **3b**.

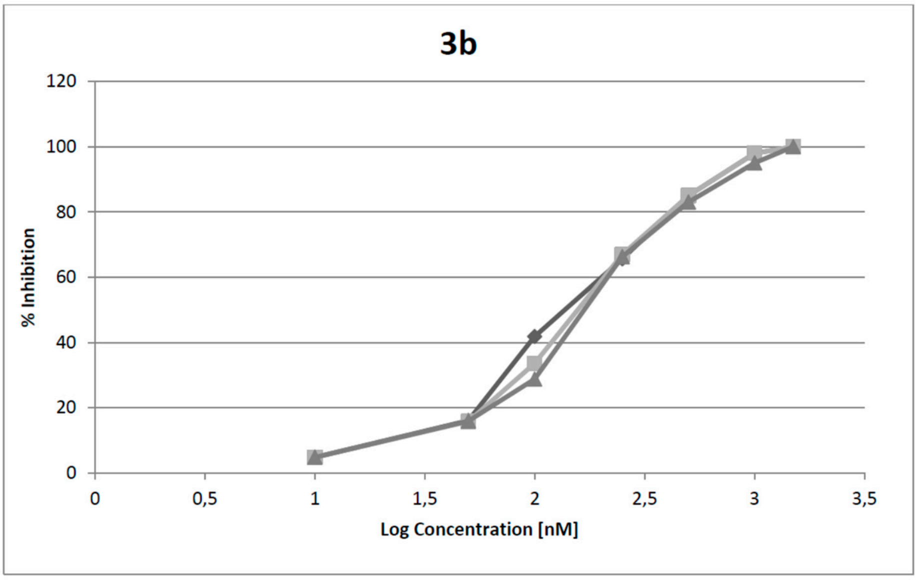

Figure S3. Curves for the determination of IC_{50} values for inhibition of HNE activity of compound **3c**.

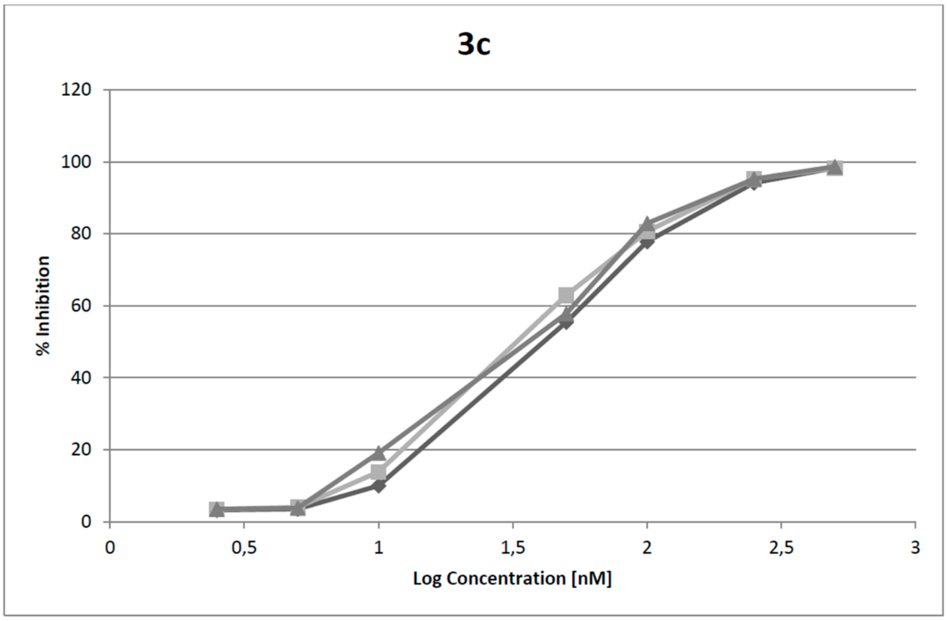

Figure S4. Curves for the determination of IC_{50} values for inhibition of HNE activity of compound **3d**.

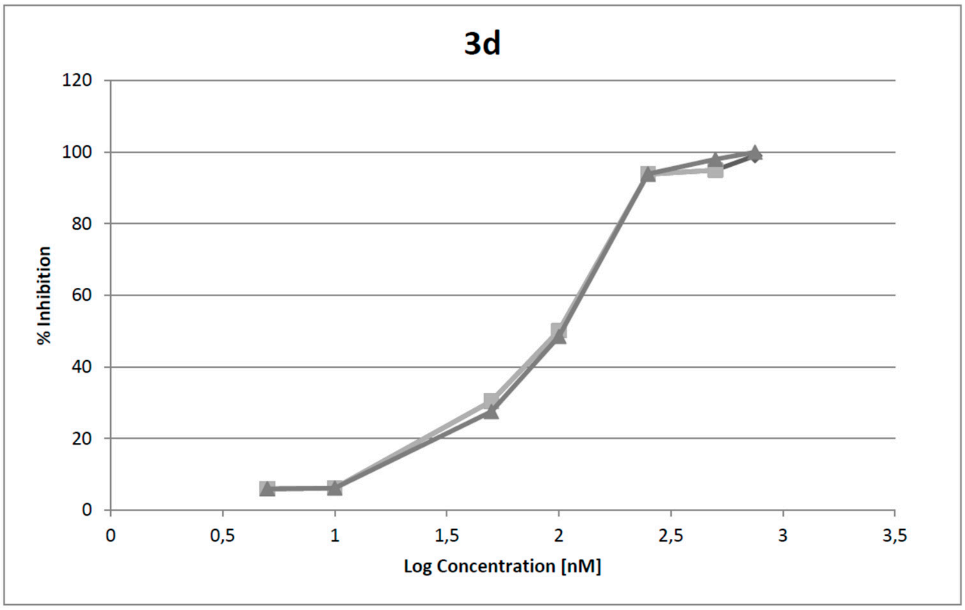

Figure S5. Curves for the determination of IC_{50} values for inhibition of HNE activity of compound **3e**.

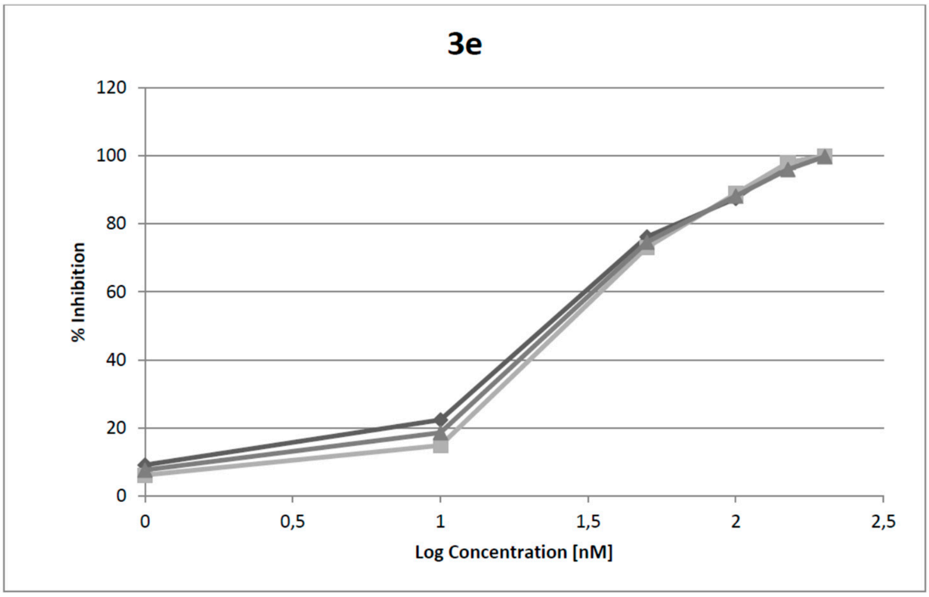

Figure S6. Curves for the determination of IC_{50} values for inhibition of HNE activity of compound **3f**.

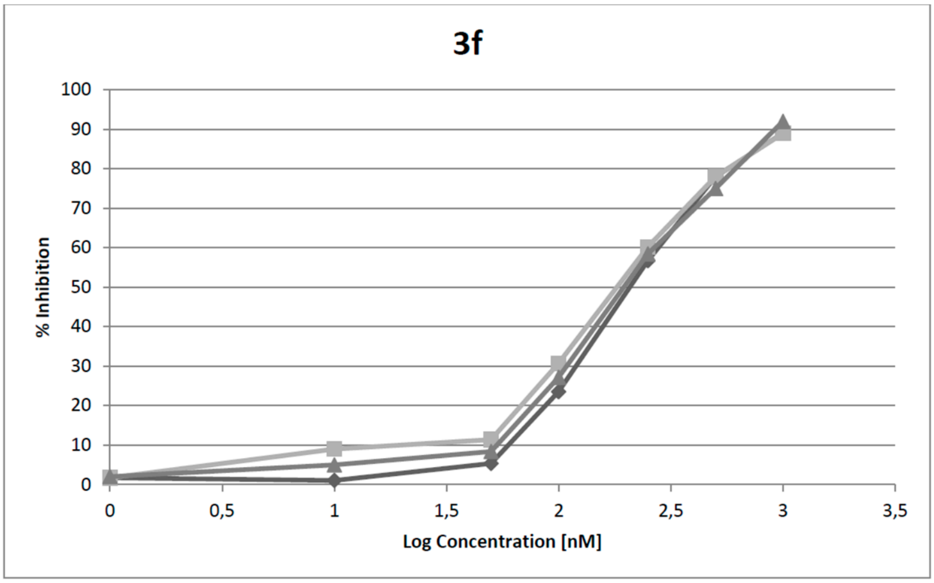

Figure S7. Curves for the determination of IC_{50} values for inhibition of HNE activity of compound **3g**.

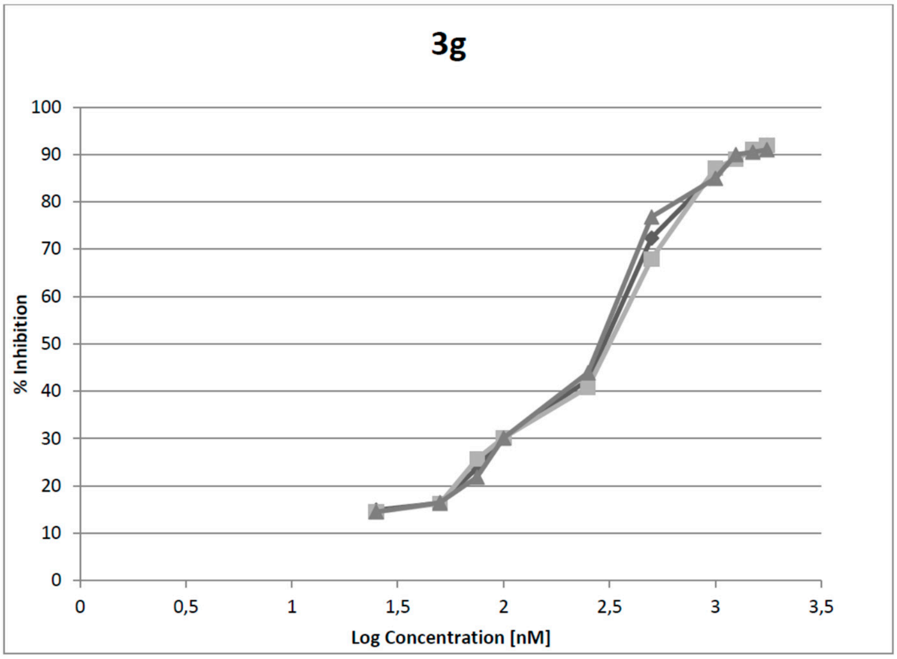

Figure S8. Curves for the determination of IC_{50} values for inhibition of HNE activity of compound **3h**.

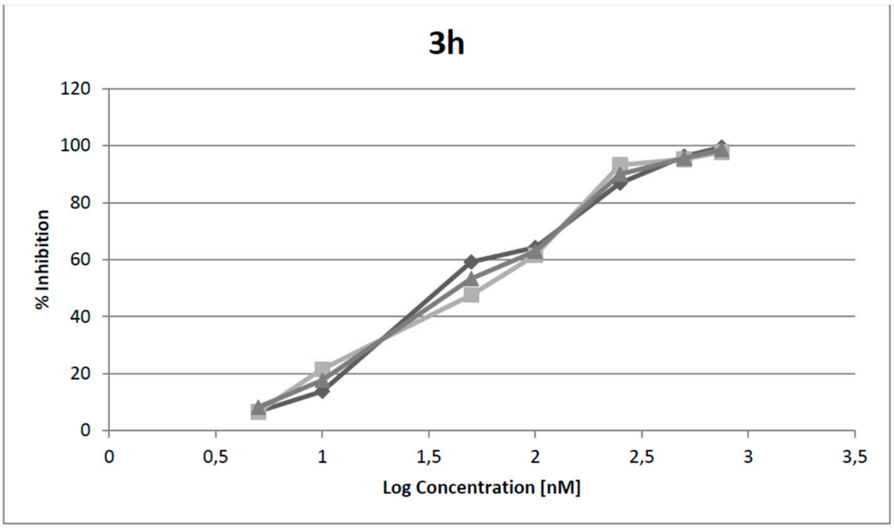

Figure S9. Curves for the determination of IC_{50} values for inhibition of HNE activity of compound **3i**.

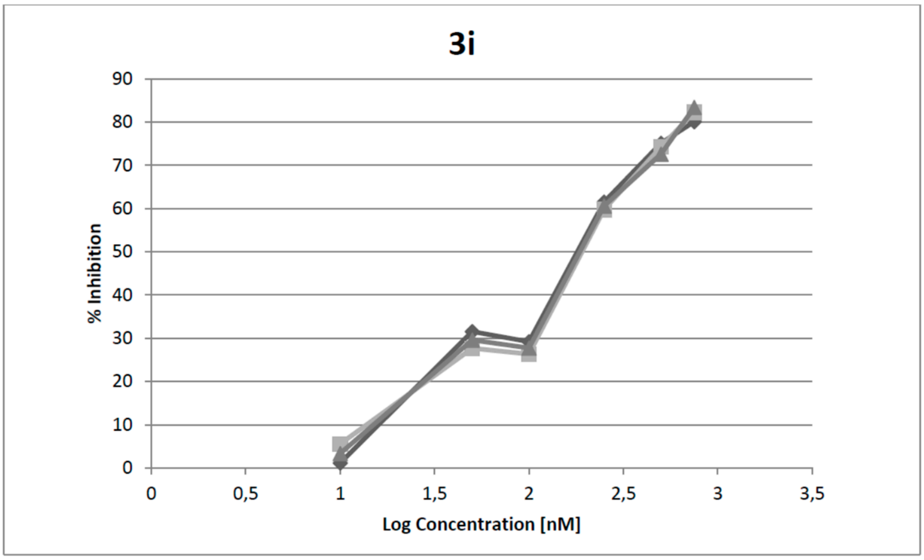

Figure S10. Curves for the determination of IC_{50} values for inhibition of HNE activity of compound **3j**.

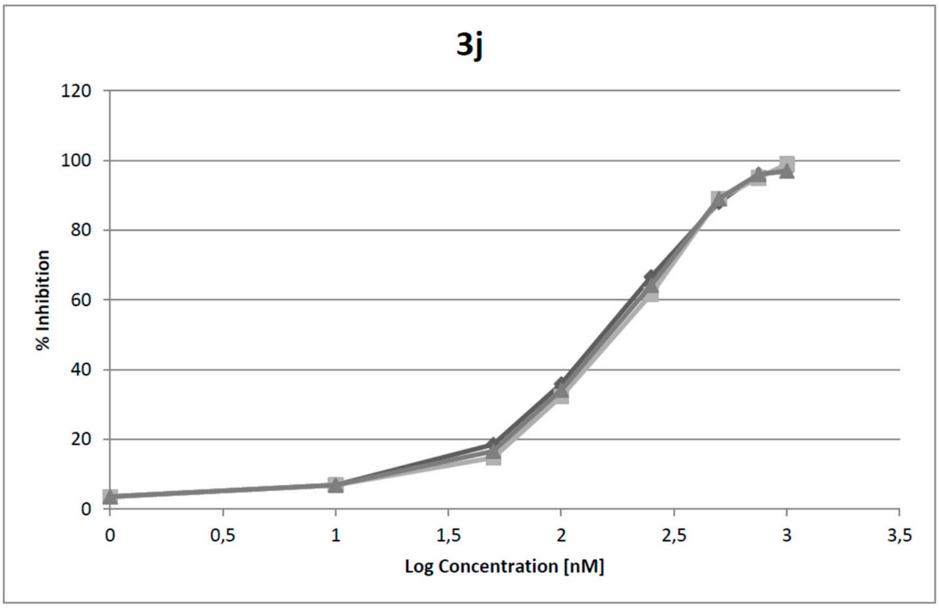

Figure S11. Curves for the determination of IC_{50} values for inhibition of HNE activity of sivelestat.

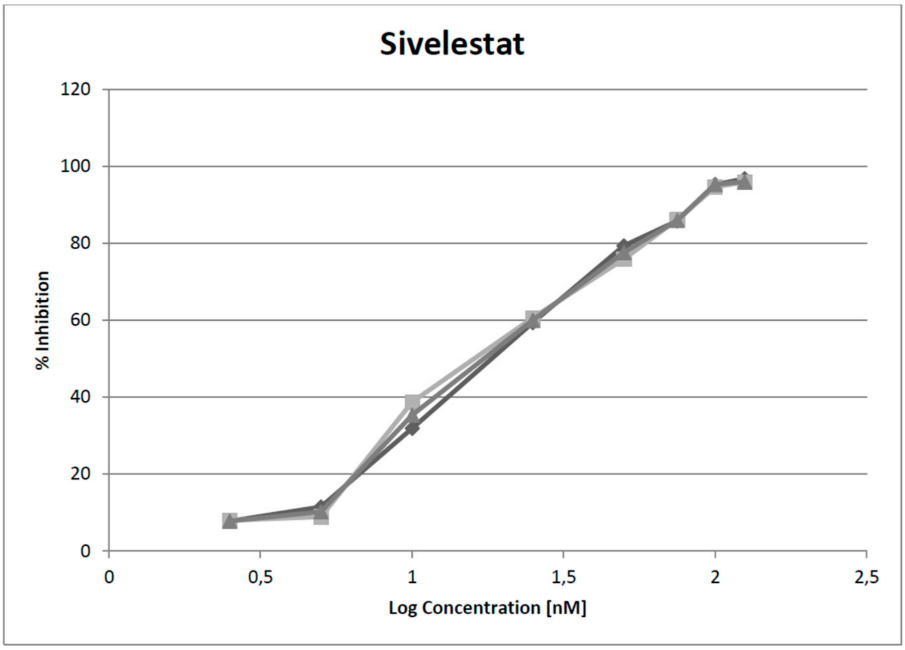

IC_{50} values obtained in [μg/ml] have been converted to [μM] values in the manuscript.

Figure S12. Curves for the determination of IC_{50} values of antiproliferative activity of compounds **3a**-**3j** for MV4-11 cancer cell line.

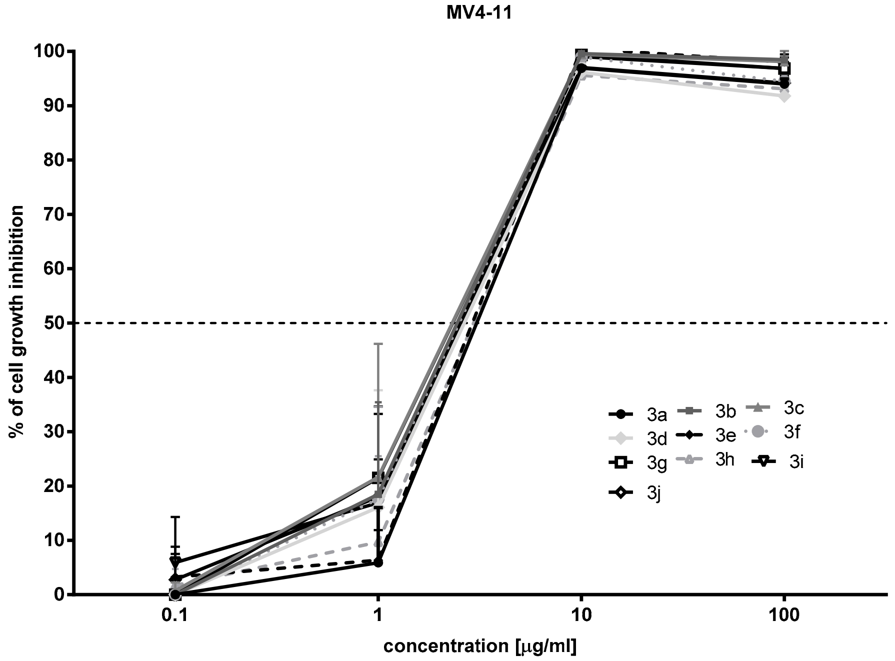

Figure S13. Curves for the determination of IC_{50} values of antiproliferative activity of compounds **3a**-**3j** for UMUC-3 cancer cell line.

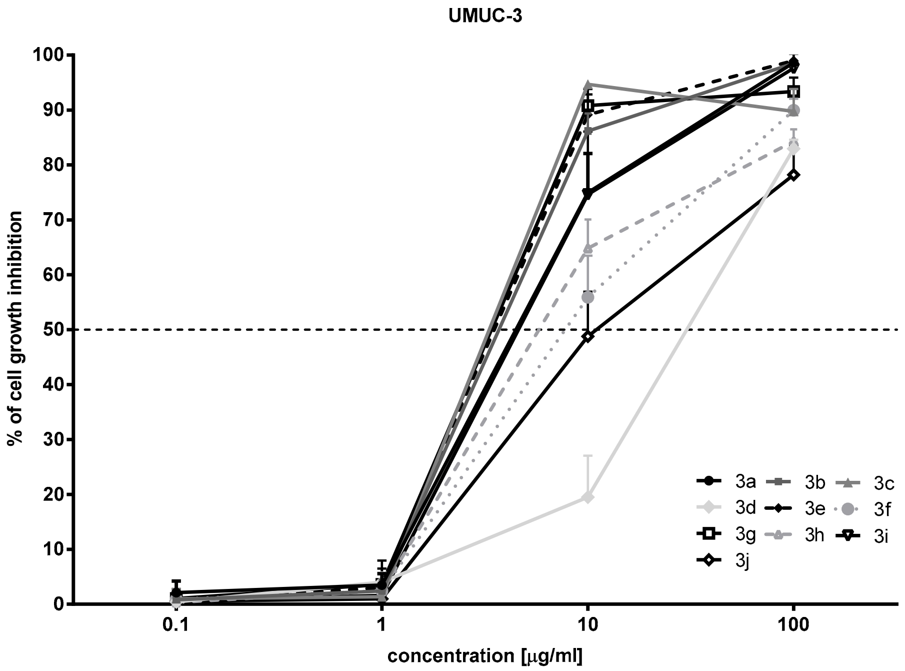

Figure S14. Curves for the determination of IC_{50} values of antiproliferative activity of compounds **3a**-**3j** for MDA-MB-231 cancer cell line.

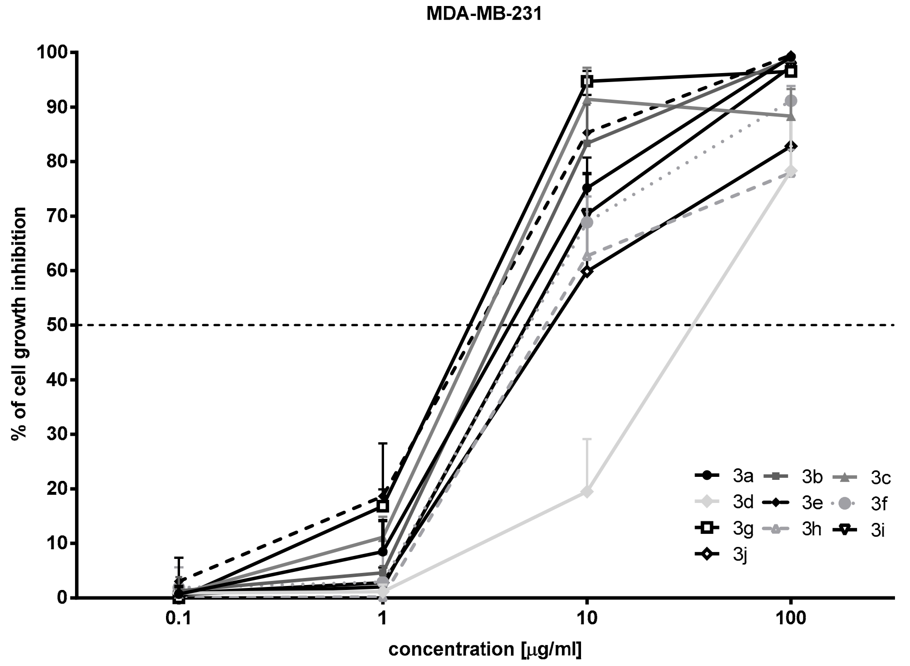

Figure S15. Curves for the determination of IC_{50} values of antiproliferative activity of compounds **3a**-**3j** for A549 cancer cell line.

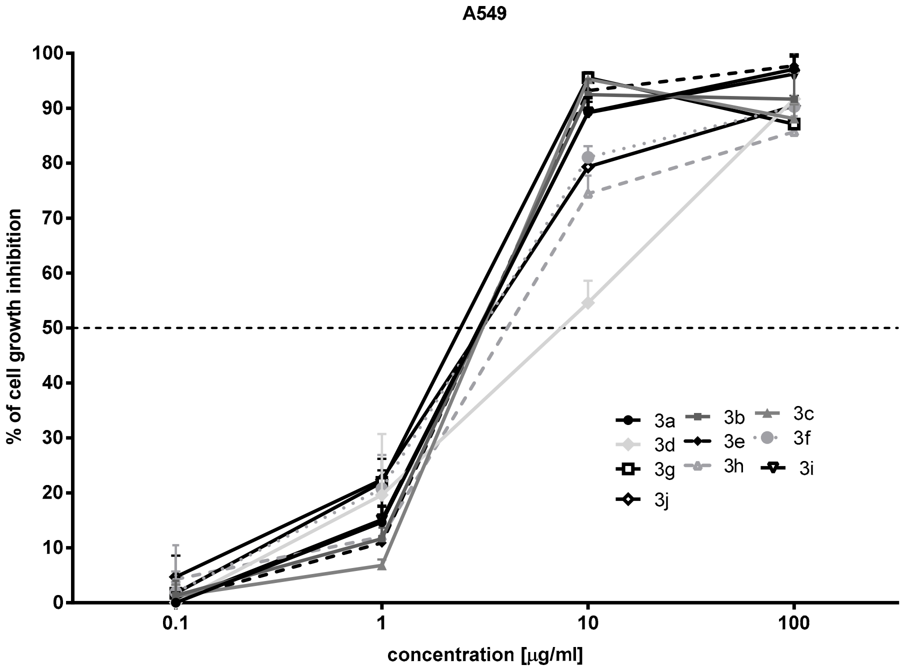

Figure S16. Curves for the determination of IC_{50} values of antiproliferative activity of compounds **3a**-**3j** for Balb/3T3 cell line.

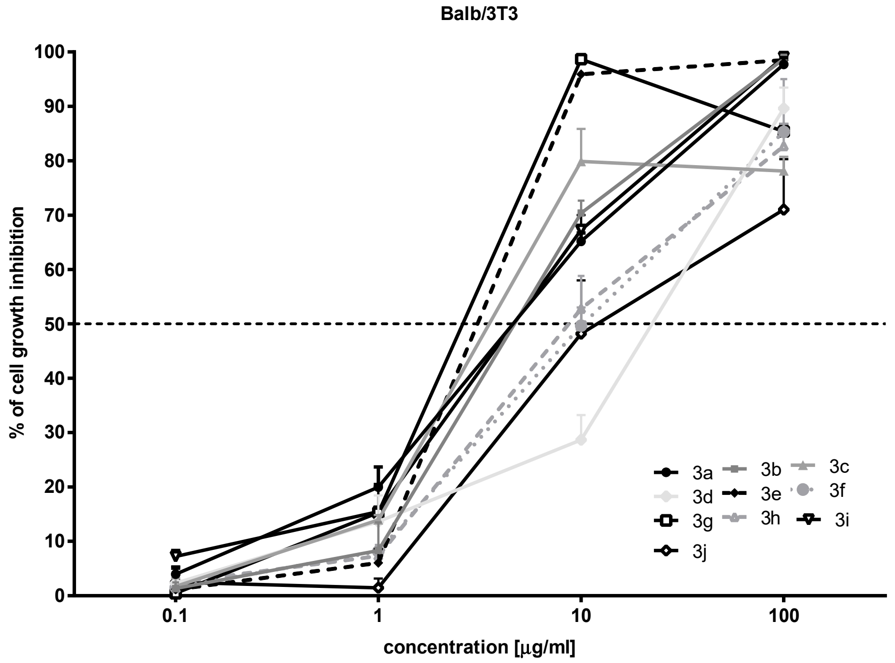

Substrate 2

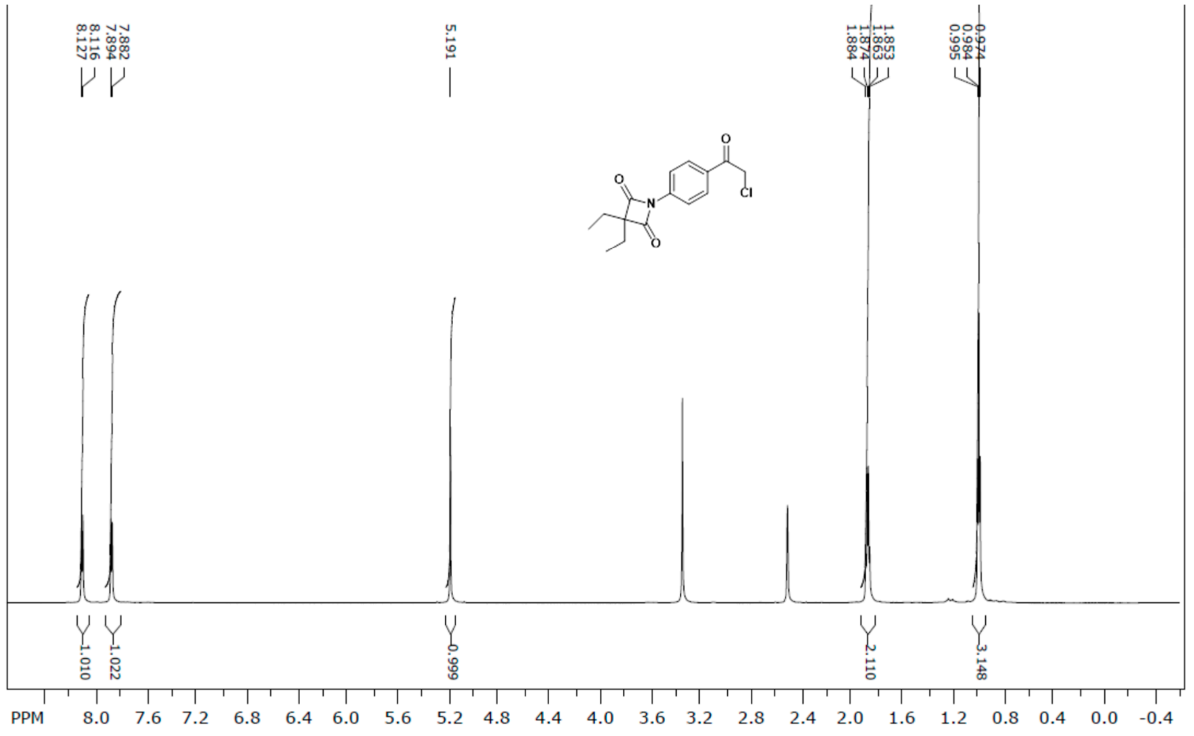

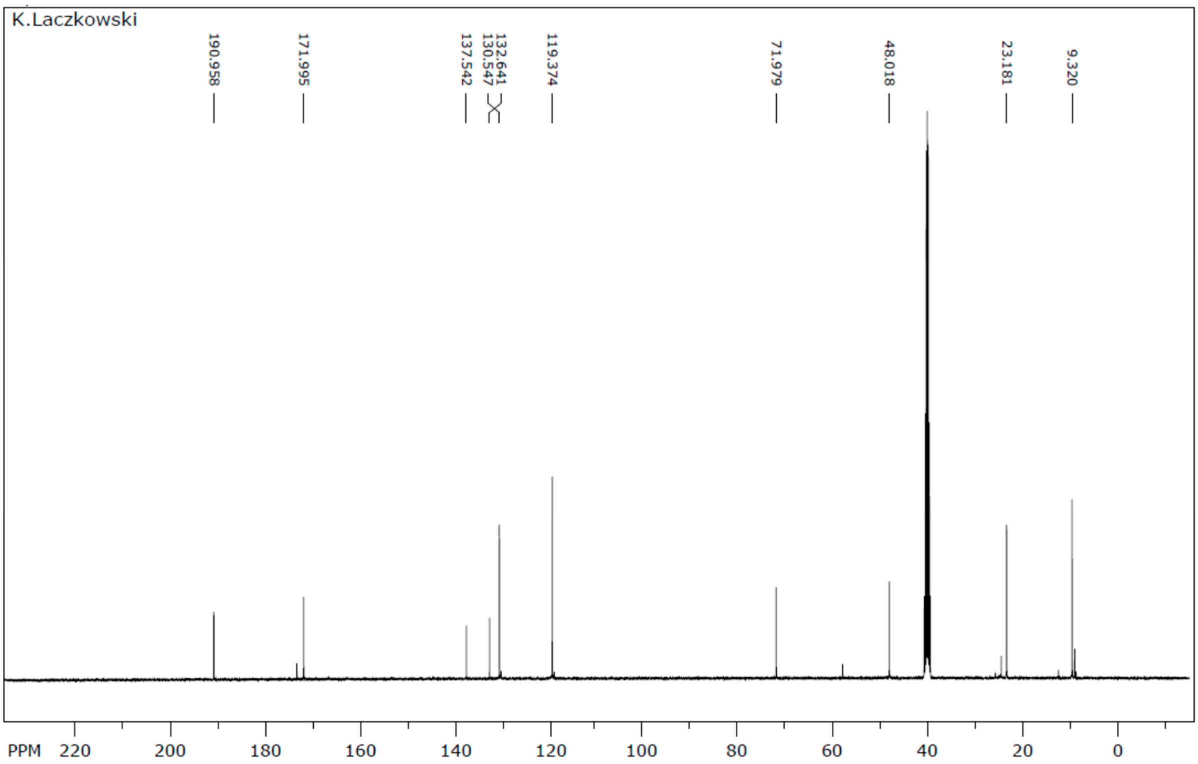
K.Laczkowski
190.958
171.995
137.542
132.641
130.547
119.374
71.979
48.018
23.181
9.320
PPM 220 200 180 160 140 120 100 80 60 40 20 0

Product 3a

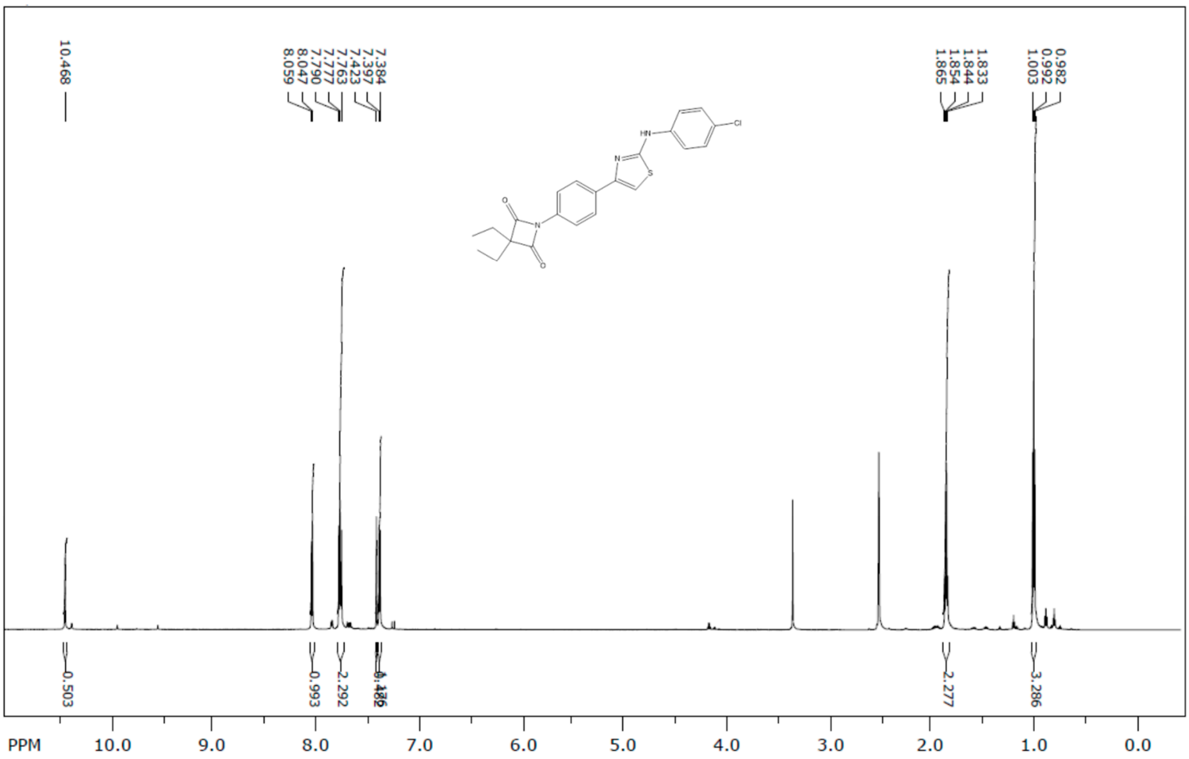
10.468
8.059
8.047
7.790
7.777
7.763
7.423
7.397
7.384
1.865
1.854
1.844
1.833
1.003
0.992
0.982
HN
Cl
N
S
O
N
O
0.503
0.993
2.292
2.277
3.286
PPM 10.0 9.0 8.0 7.0 6.0 5.0 4.0 3.0 2.0 1.0 0.0

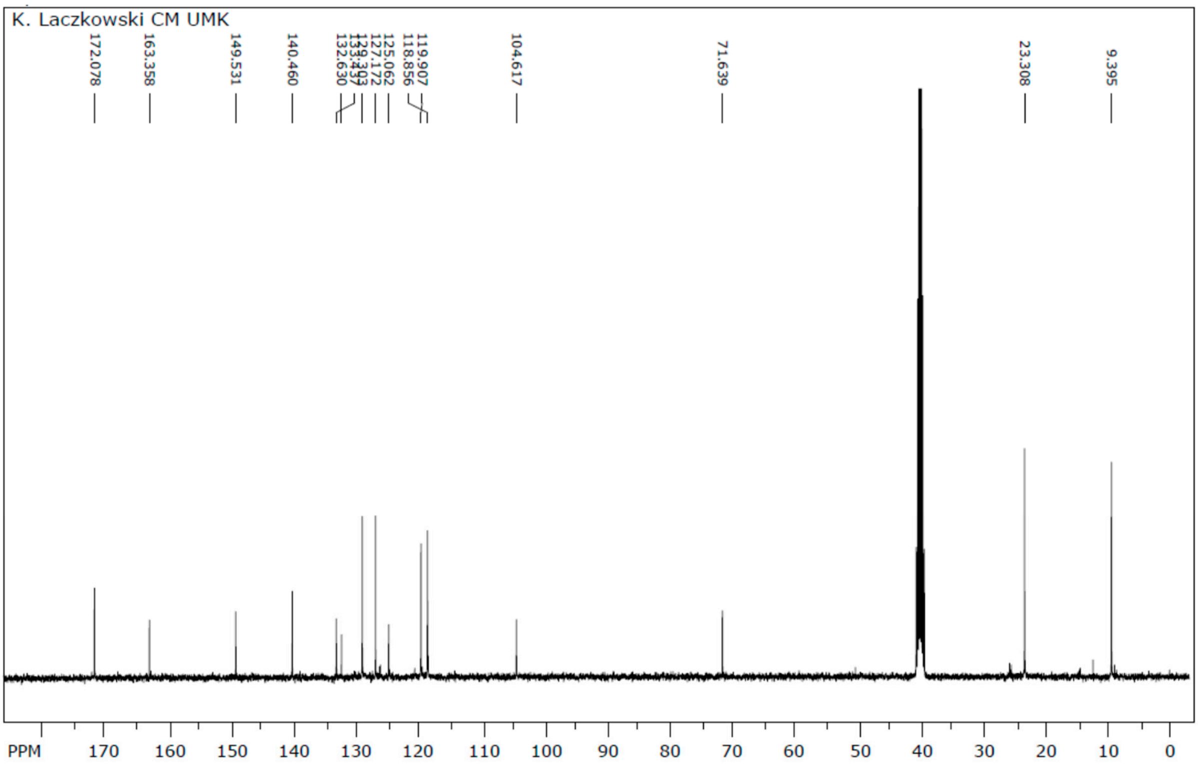
K. Laczkowski CM UMK
172.078
163.358
149.531
140.460
132.630
127.172
125.062
118.856
119.907
104.617
71.639
23.308
9.395
PPM
170
160
150
140
130
120
110
100
90
80
70
60
50
40
30
20
10
0

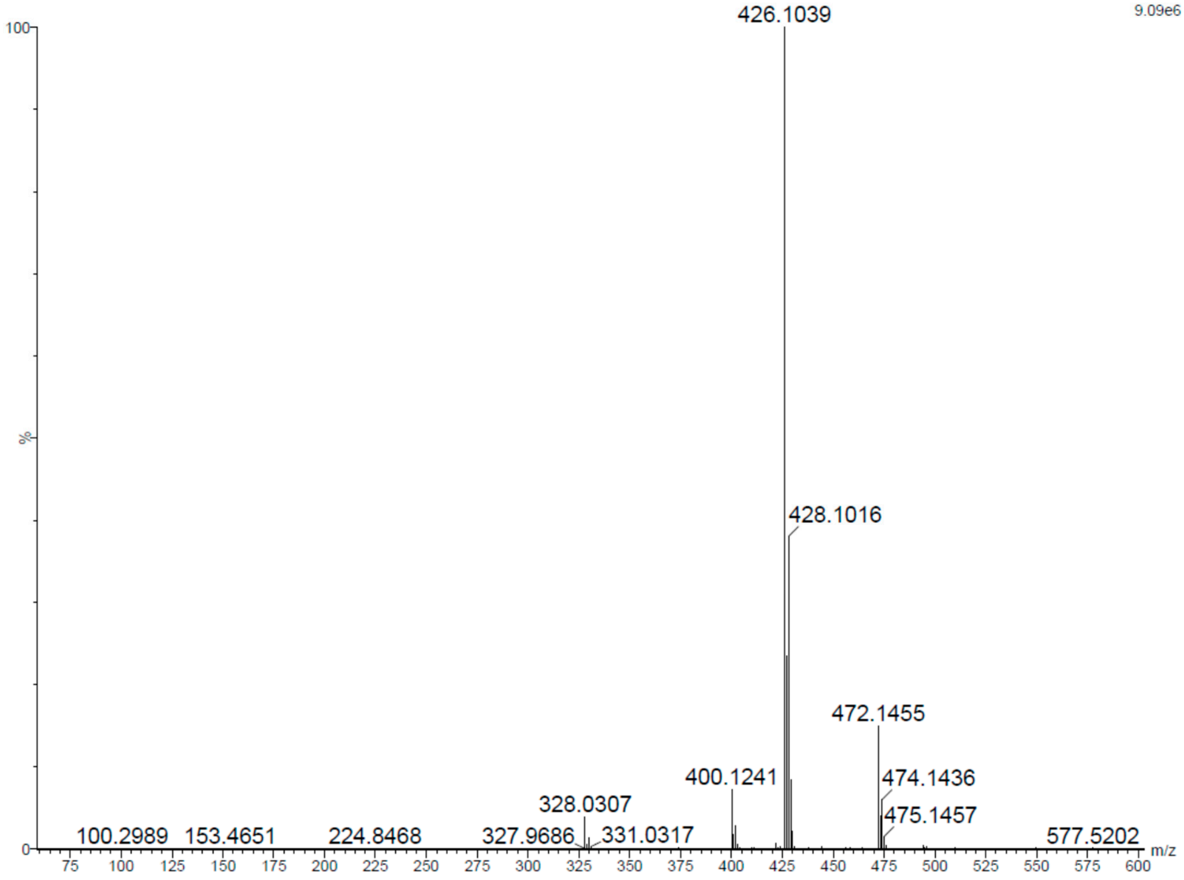
426.1039
9.09e6
100
%
0
428.1016
472.1455
474.1436
475.1457
400.1241
328.0307
331.0317
327.9686
100.2989
153.4651
224.8468
577.5202
m/z
75
100
125
150
175
200
225
250
275
300
325
350
375
400
425
450
475
500
525
550
575
600

Product 3b

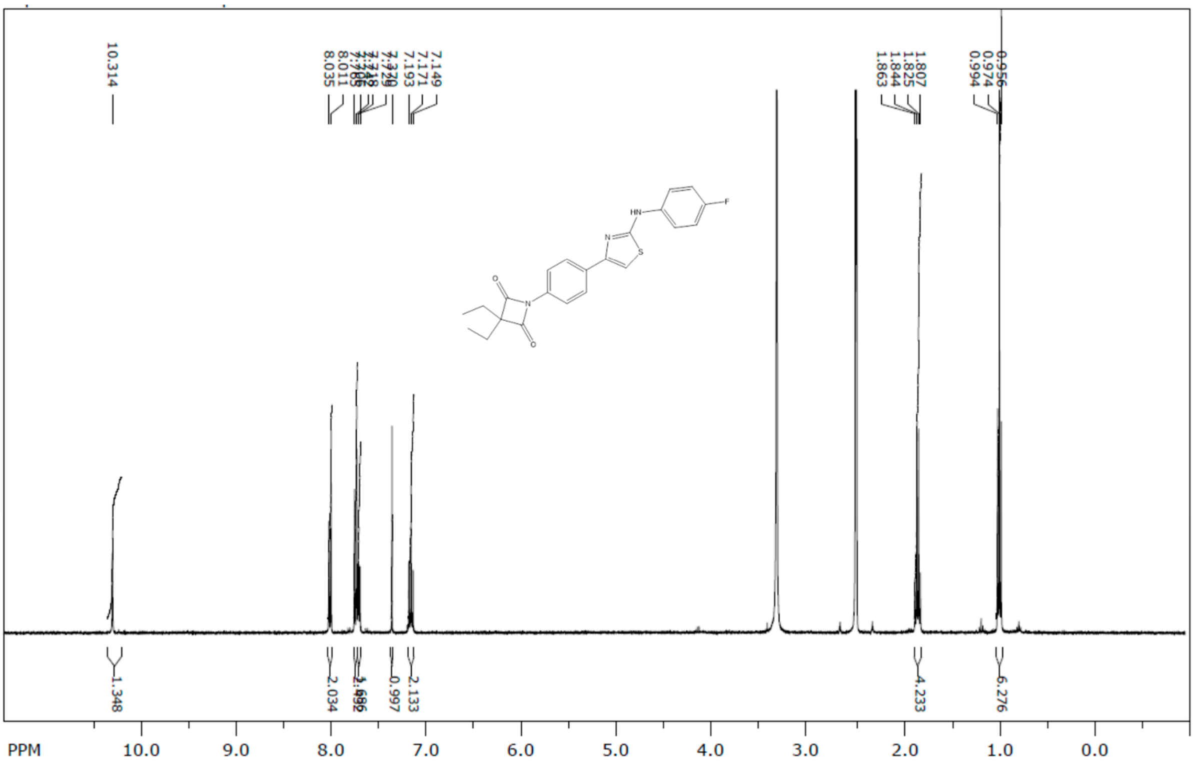

10.314
8.035
8.011
7.193
7.171
7.149
1.863
1.844
1.825
1.807
0.994
0.974
0.956
HN
F
N
S
N
O
O
1.348
2.034
0.997
2.133
4.233
6.276
PPM
10.0
9.0
8.0
7.0
6.0
5.0
4.0
3.0
2.0
1.0
0.0

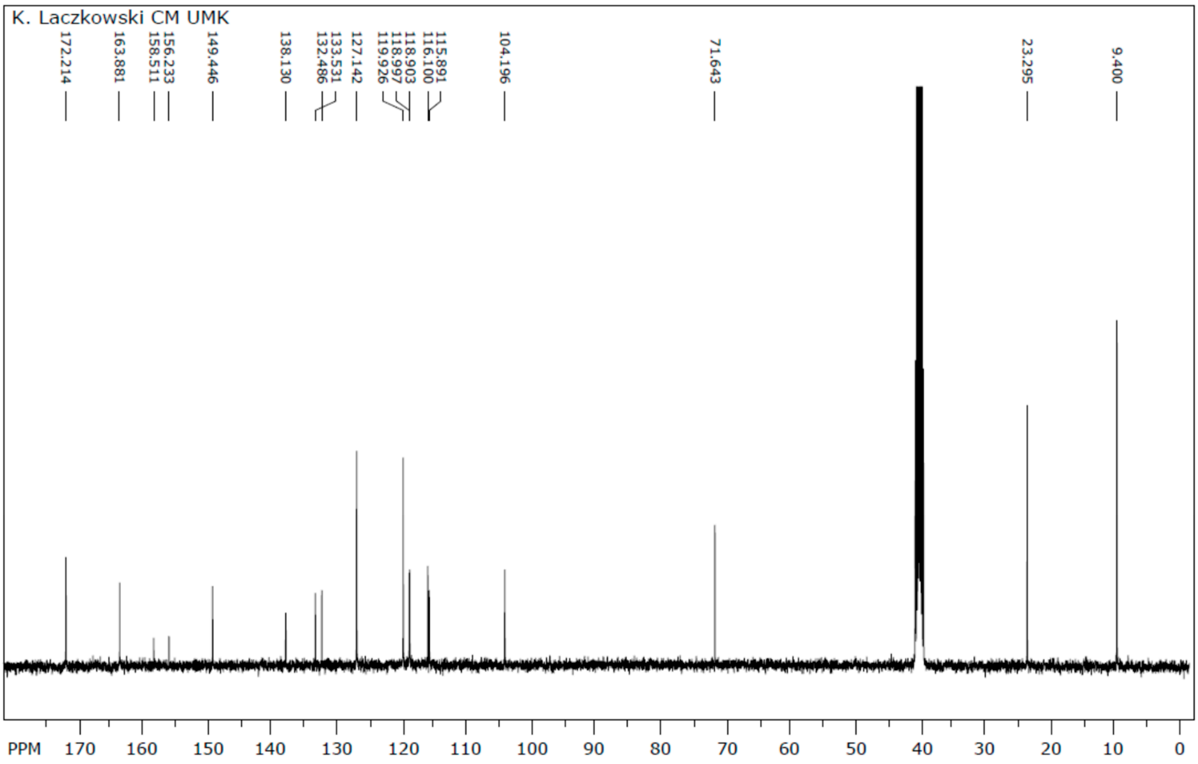

K. Laczkowski CM UMK
172.214
163.881
158.511
156.233
149.446
138.130
133.531
132.486
127.142
119.926
118.997
118.903
116.100
115.891
104.196
71.643
23.295
9.400
PPM
170
160
150
140
130
120
110
100
90
80
70
60
50
40
30
20
10
0

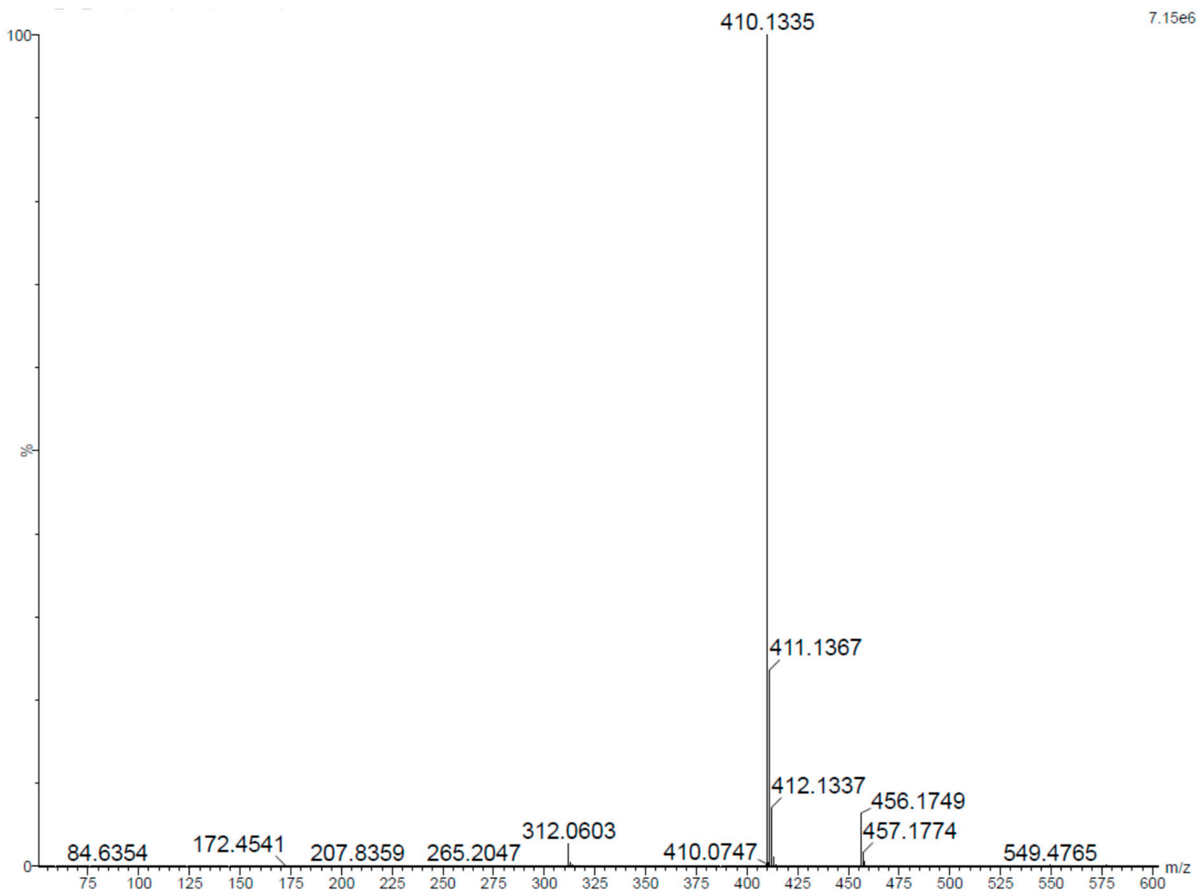
410.1335
7.15e6
100
%
0
411.1367
412.1337
456.1749
457.1774
312.0603
84.6354
172.4541
207.8359
265.2047
410.0747
549.4765
m/z

Product 3c

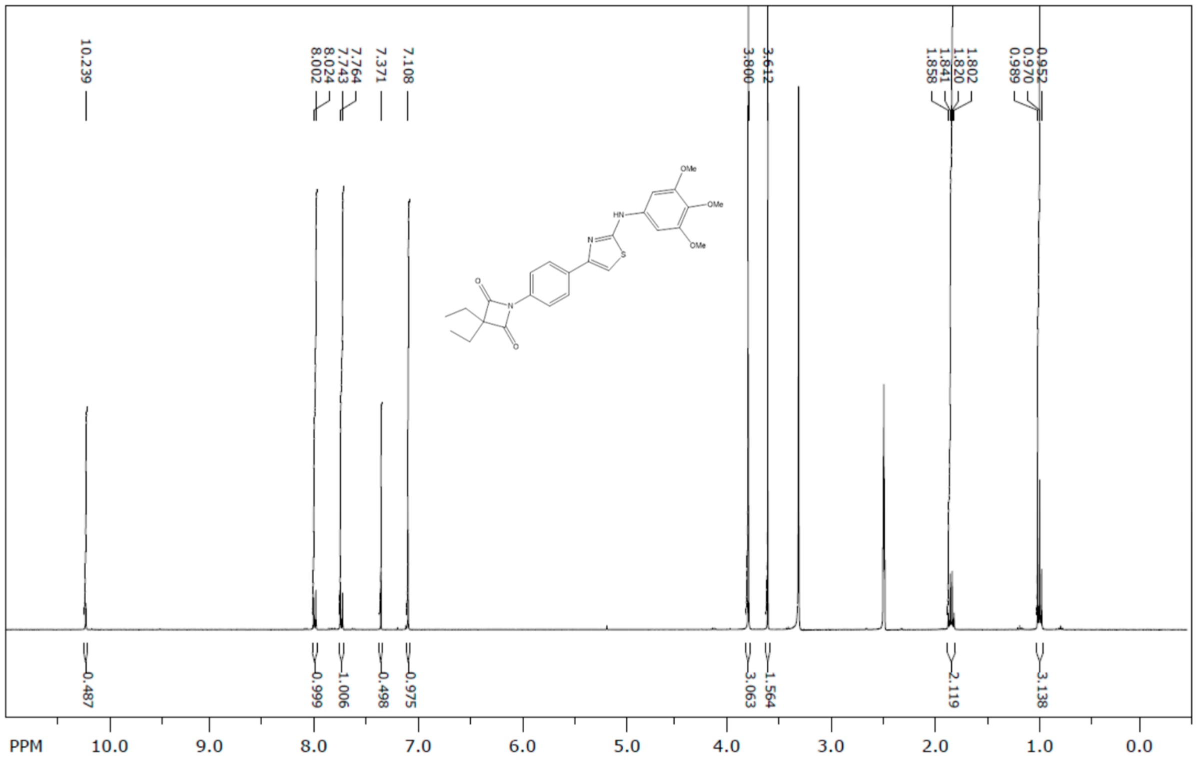
10.239
8.024
8.002
7.764
7.743
7.371
7.108
3.800
3.612
1.858
1.841
1.820
1.802
0.989
0.970
0.952
0.487
0.999
1.006
0.498
0.975
3.063
1.564
2.119
3.138
PPM
OMe
HN
N
S
O

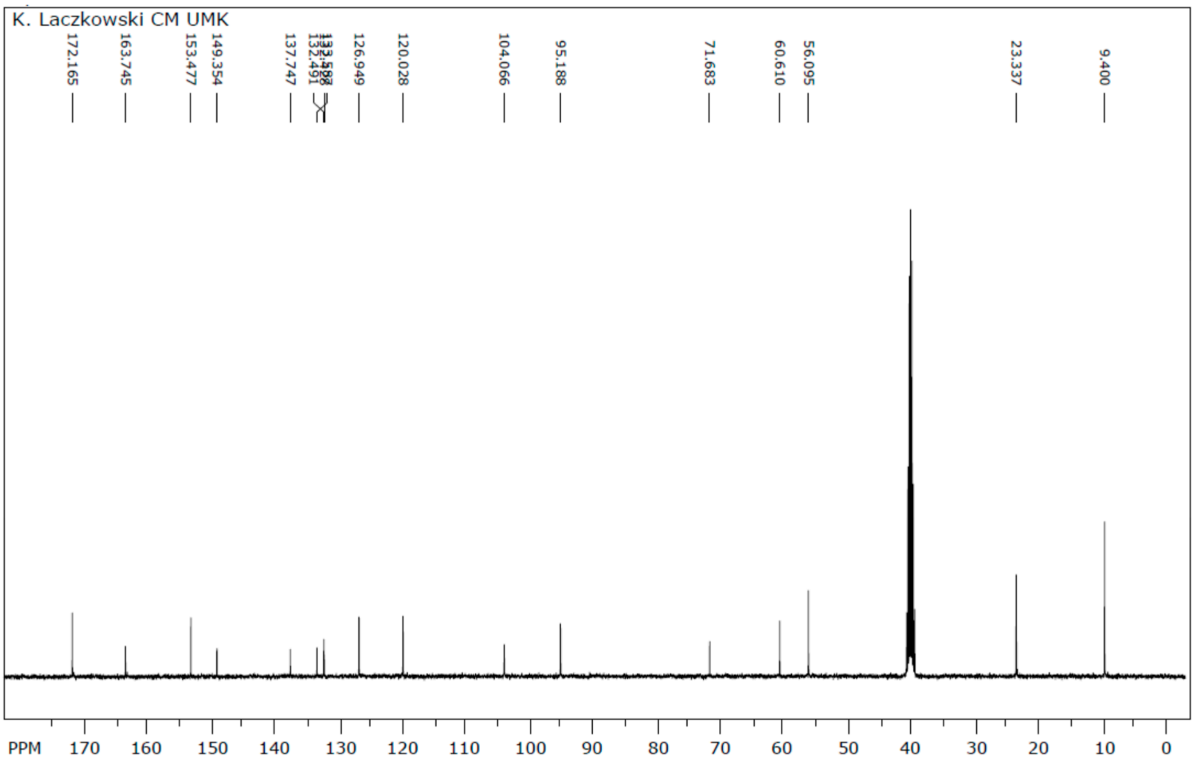
K. Laczkowski CM UMK
172.165
163.745
153.477
149.354
137.747
132.491
126.949
120.028
104.066
95.188
71.683
60.610
56.095
23.337
9.400
PPM
170
160
150
140
130
120
110
100
90
80
70
60
50
40
30
20
10
0

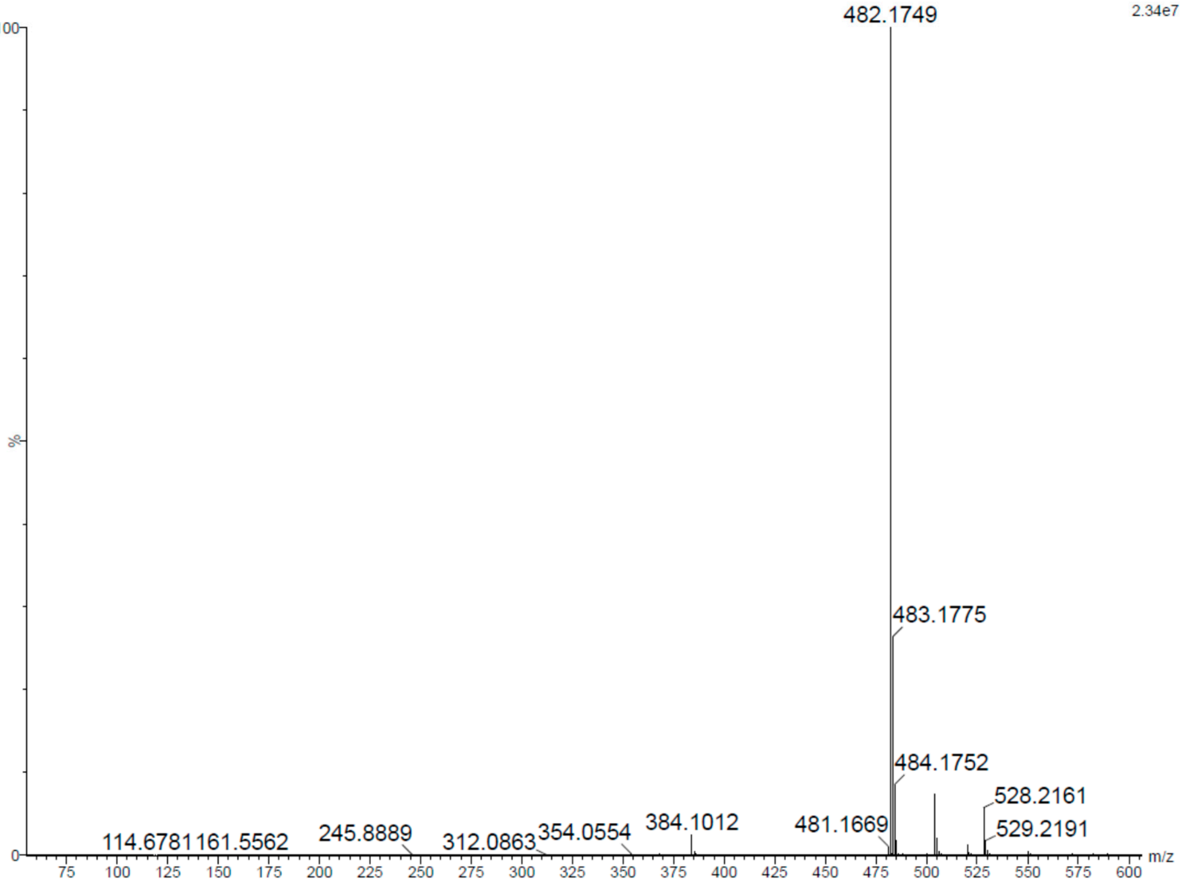
482.1749
2.34e7
100
%
0
483.1775
484.1752
528.2161
529.2191
481.1669
384.1012
354.0554
312.0863
245.8889
114.6781
161.5562
m/z
75
100
125
150
175
200
225
250
275
300
325
350
375
400
425
450
475
500
525
550
575
600

Product 3d

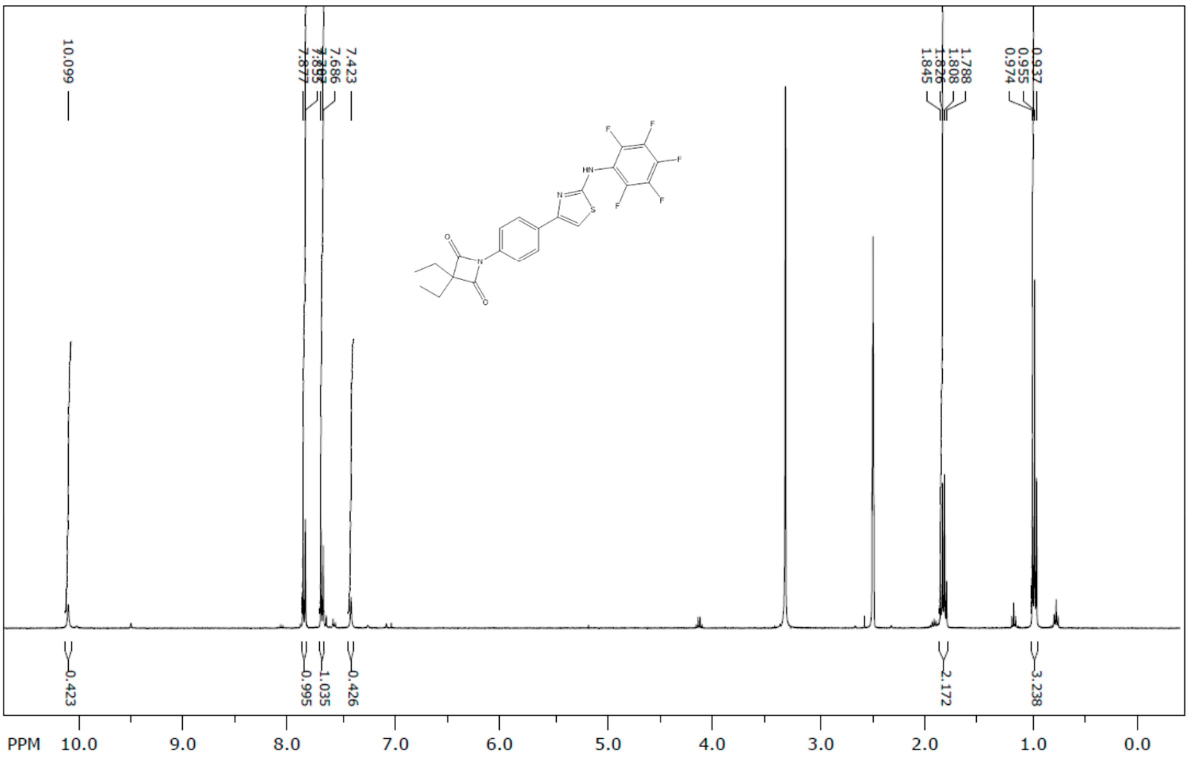
10.099
7.877
7.686
7.423
1.845
1.826
1.808
1.788
0.974
0.955
0.937
0.423
0.995
1.035
0.426
2.172
3.238
PPM 10.0 9.0 8.0 7.0 6.0 5.0 4.0 3.0 2.0 1.0 0.0

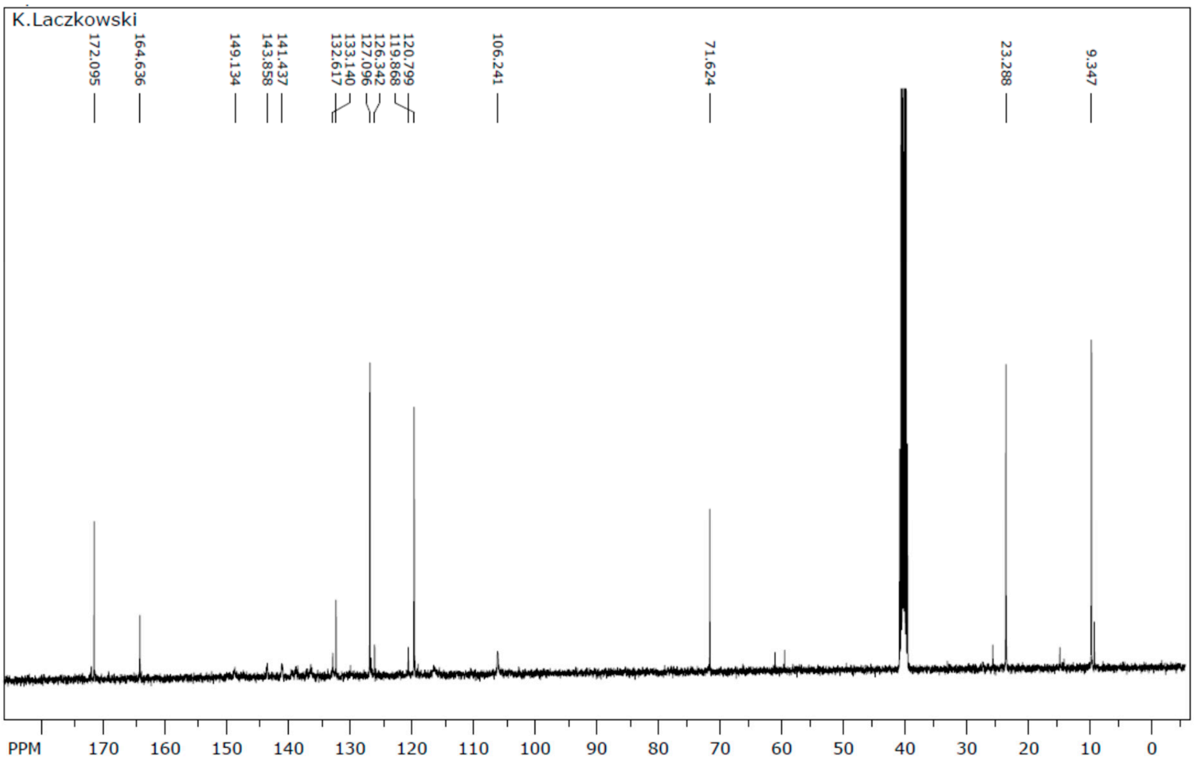
K.Laczkowski
172.095
164.636
149.134
143.858
141.437
133.140
132.617
127.096
126.342
120.799
119.868
106.241
71.624
23.288
9.347
PPM 170 160 150 140 130 120 110 100 90 80 70 60 50 40 30 20 10 0

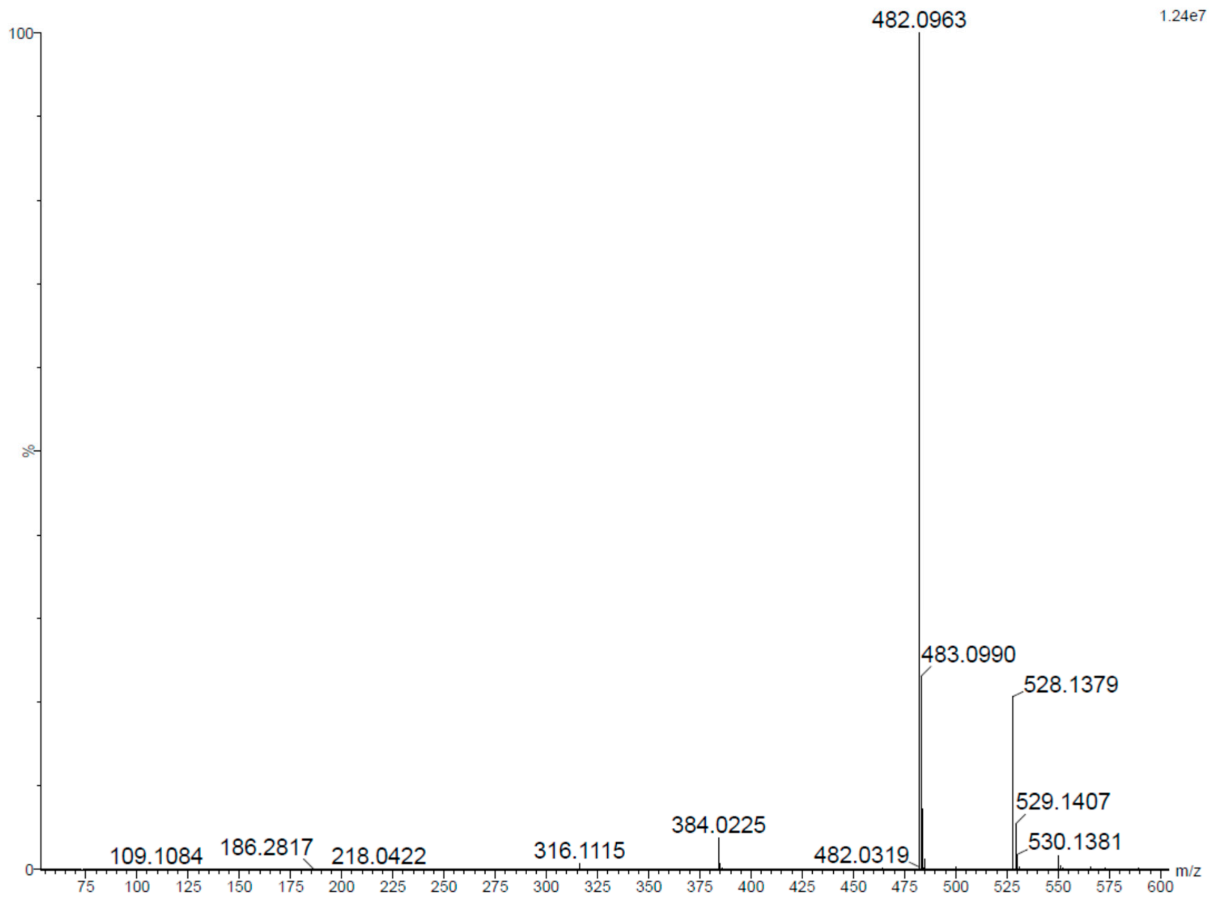
482.0963
1.24e7
100
%
0
483.0990
528.1379
529.1407
530.1381
384.0225
482.0319
316.1115
218.0422
186.2817
109.1084
m/z

Product 3e

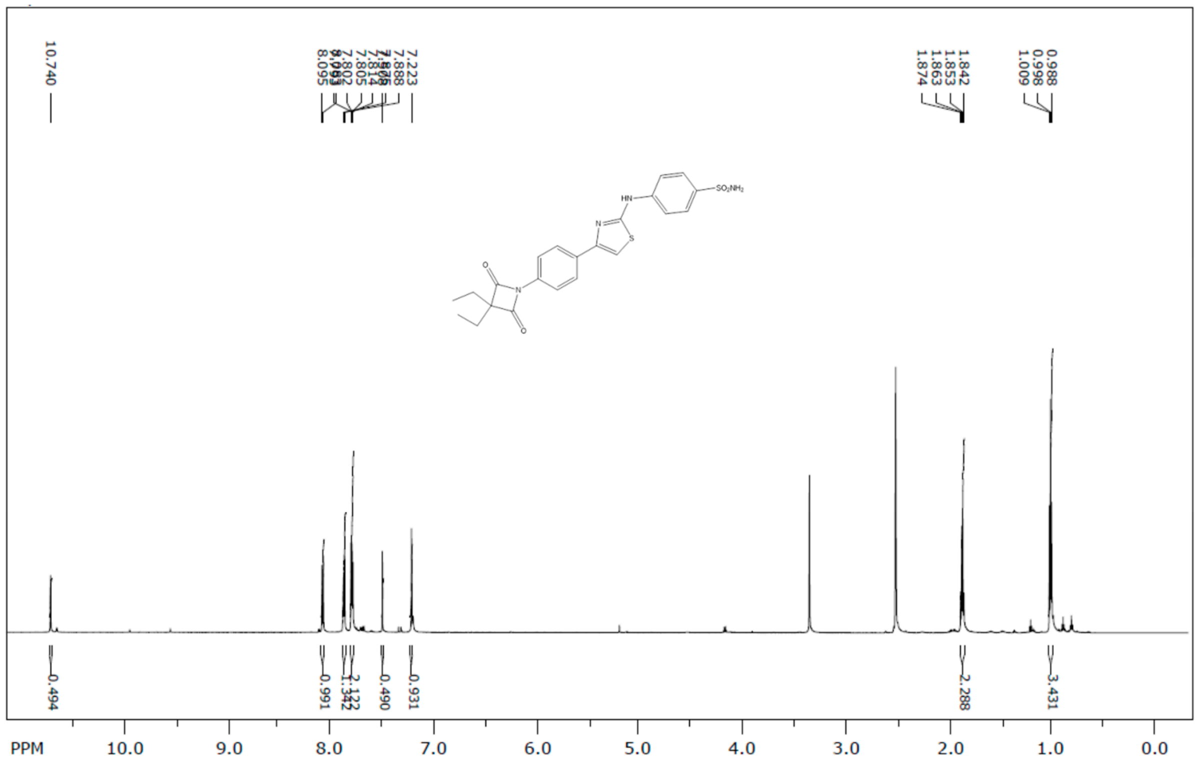
10.740
8.095
7.802
7.805
7.888
7.223
1.874
1.863
1.853
1.842
1.009
0.998
0.988
0.494
0.991
0.490
0.931
2.288
3.431
PPM
SO2NH2
HN
N
S
O
N

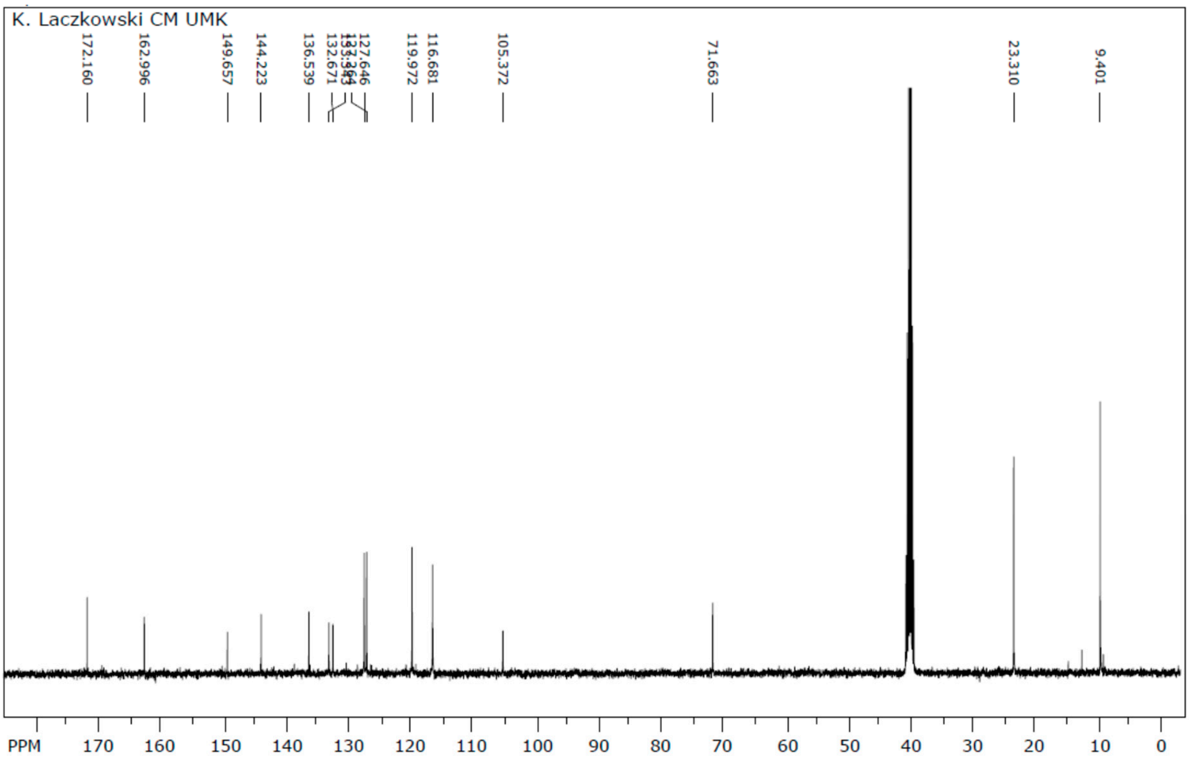
K. Laczkowski CM UMK
172.160
162.996
149.657
144.223
136.539
132.671
127.646
119.972
116.681
105.372
71.663
23.310
9.401
PPM
170 160 150 140 130 120 110 100 90 80 70 60 50 40 30 20 10 0

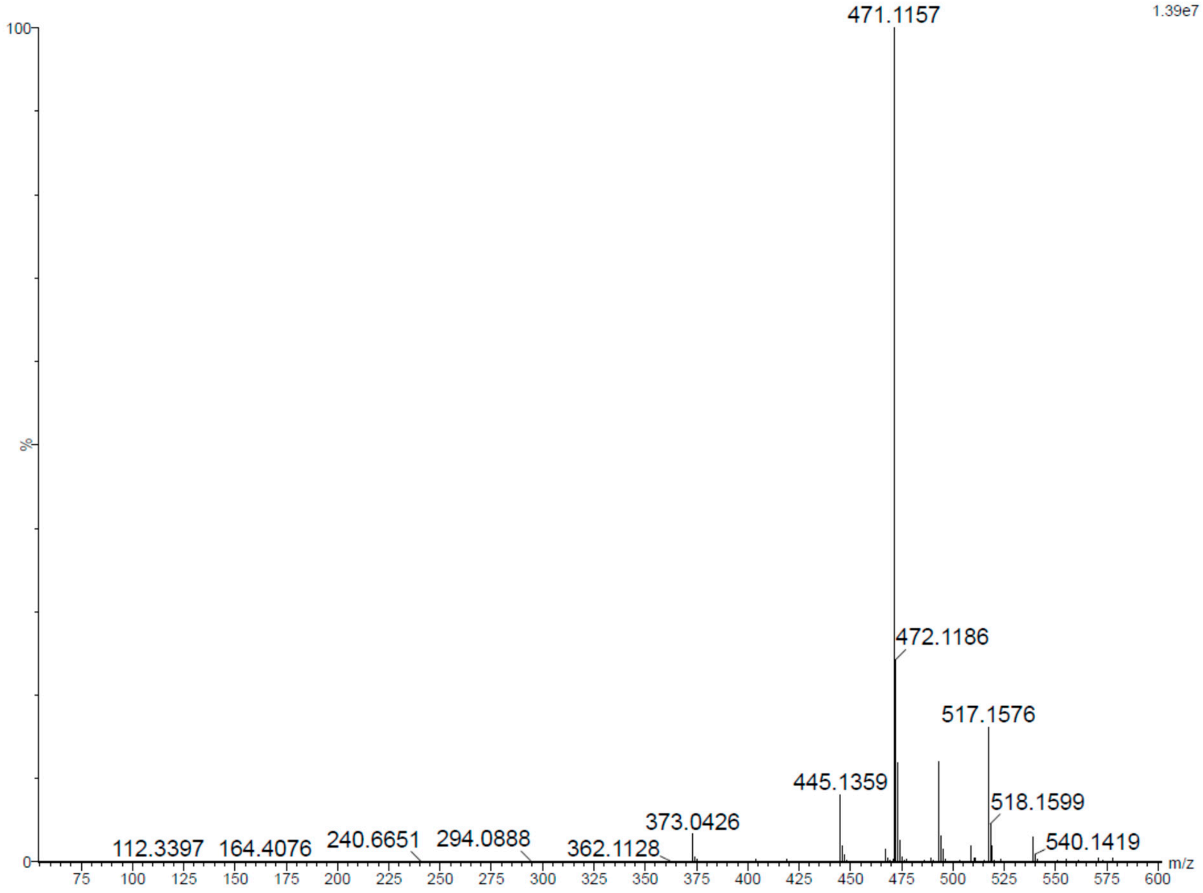
471.1157
1.39e7
100
%
0
472.1186
517.1576
445.1359
518.1599
373.0426
540.1419
112.3397
164.4076
240.6651
294.0888
362.1128
m/z
75 100 125 150 175 200 225 250 275 300 325 350 375 400 425 450 475 500 525 550 575 600

Product 3f

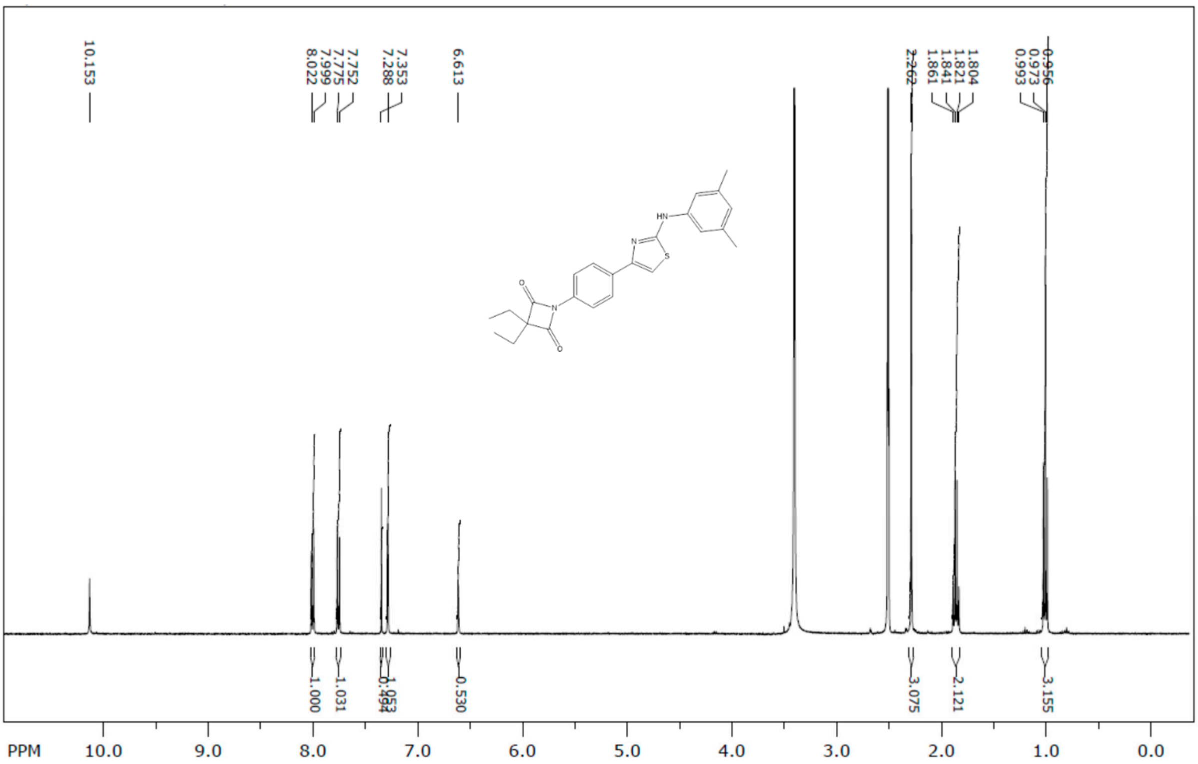

10.153
8.022
7.999
7.775
7.752
7.353
7.288
6.613
2.262
1.861
1.841
1.821
1.804
0.993
0.973
0.956
1.000
1.031
1.053
0.494
0.530
3.075
2.121
3.155
HN
N
S
O
N
O
PPM
10.0
9.0
8.0
7.0
6.0
5.0
4.0
3.0
2.0
1.0
0.0

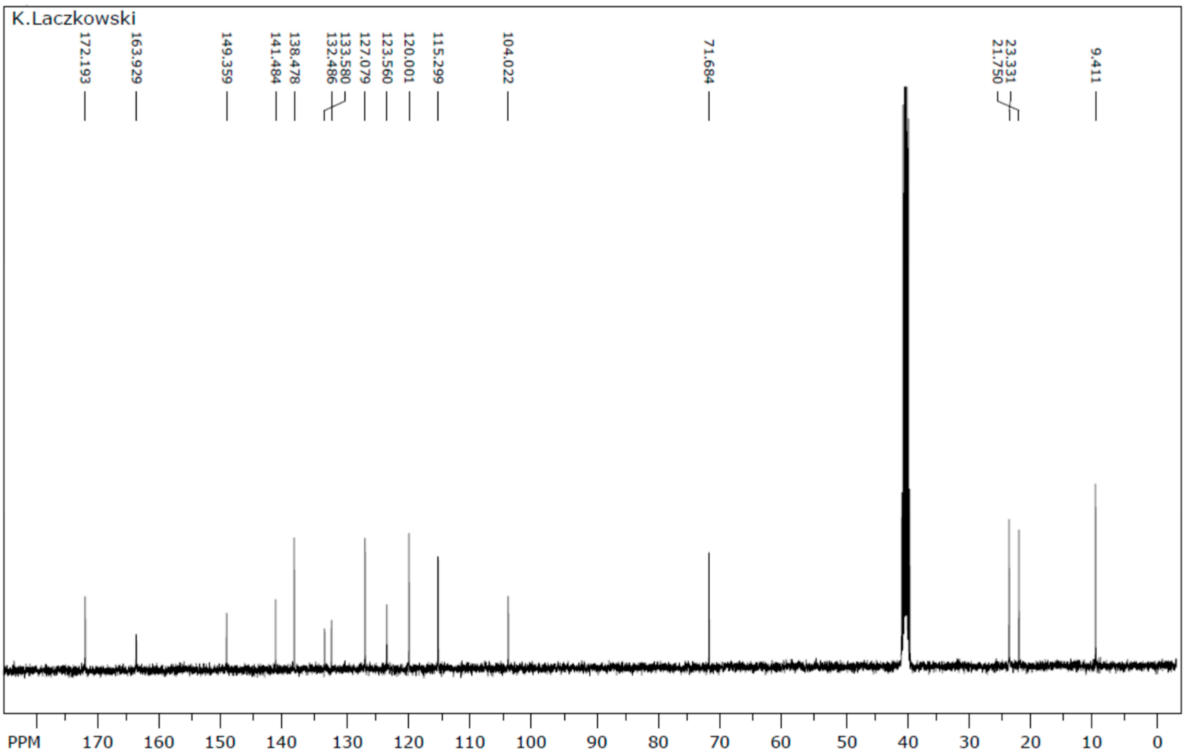

K.Laczkowski
172.193
163.929
149.359
141.484
138.478
133.580
132.486
127.079
123.560
120.001
115.299
104.022
71.684
23.331
21.750
9.411
PPM
170
160
150
140
130
120
110
100
90
80
70
60
50
40
30
20
10
0

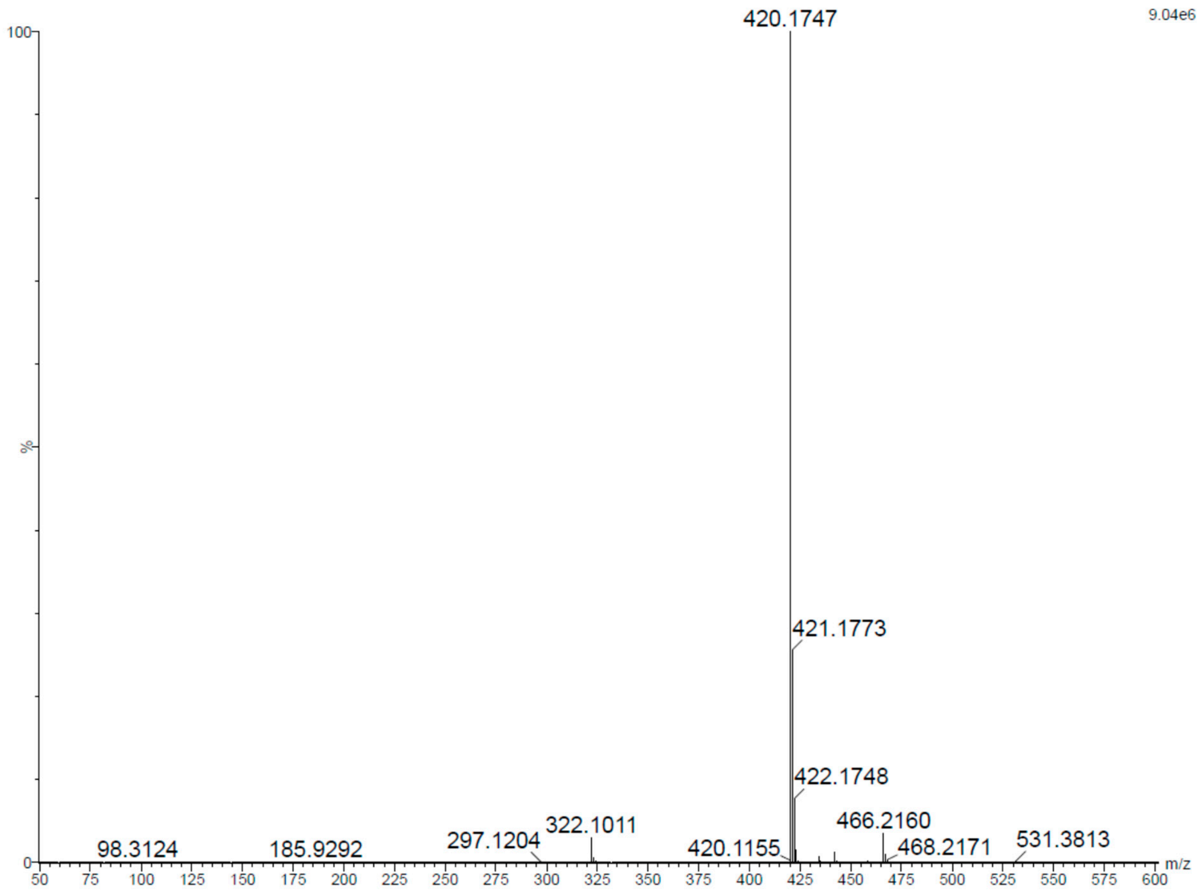

Product 3g

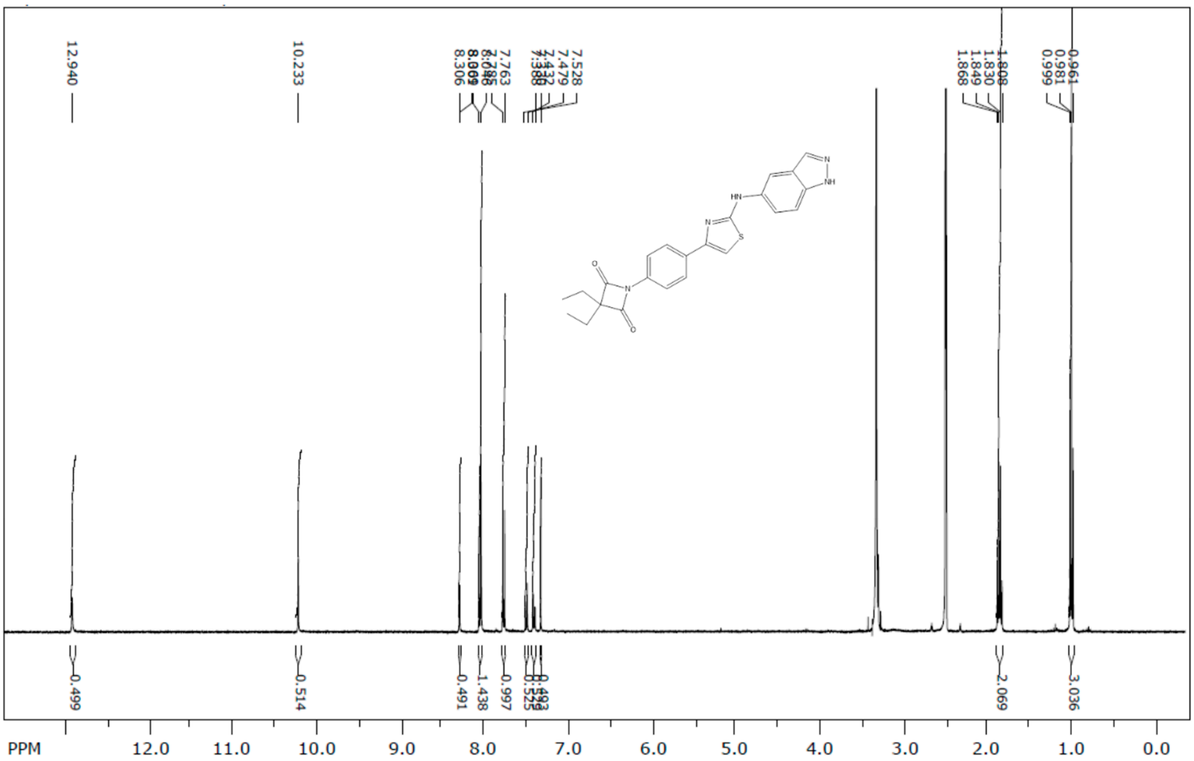

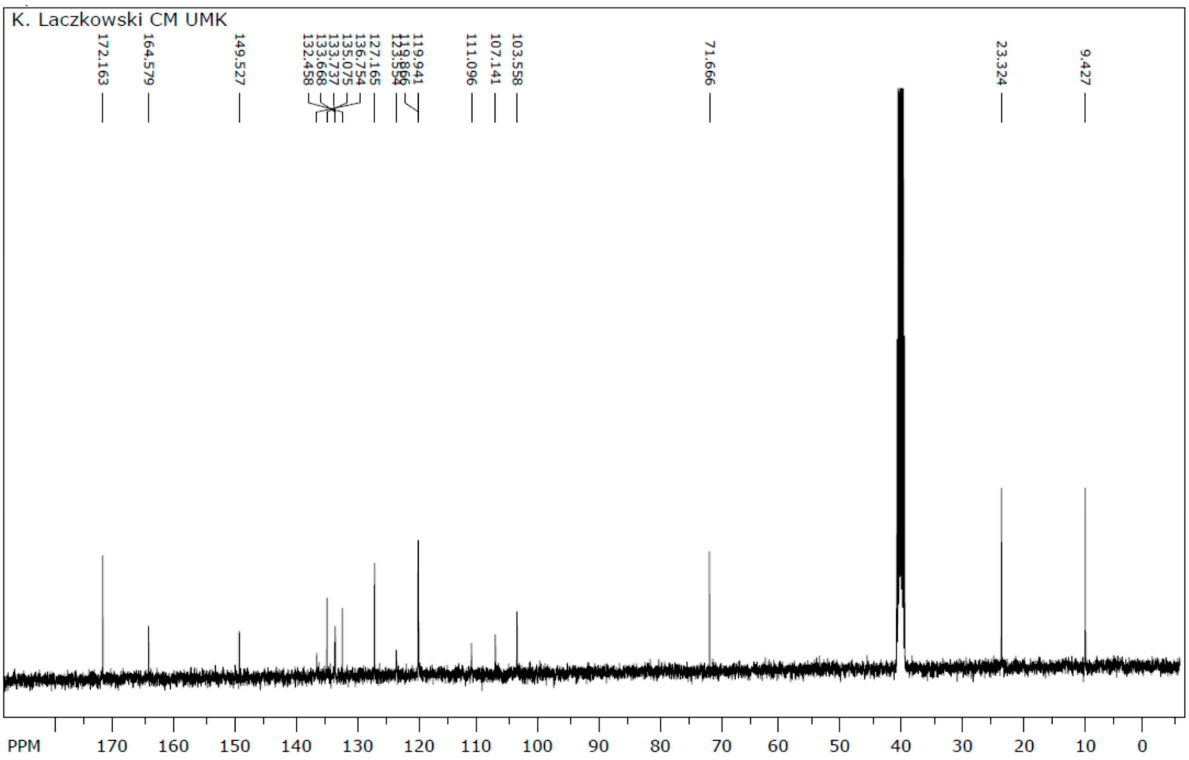
K. Laczkowski CM UMK
172.163
164.579
149.527
132.458
133.668
133.737
135.075
136.754
127.165
119.941
111.096
107.141
103.558
71.666
23.324
9.427
PPM
170
160
150
140
130
120
110
100
90
80
70
60
50
40
30
20
10
0

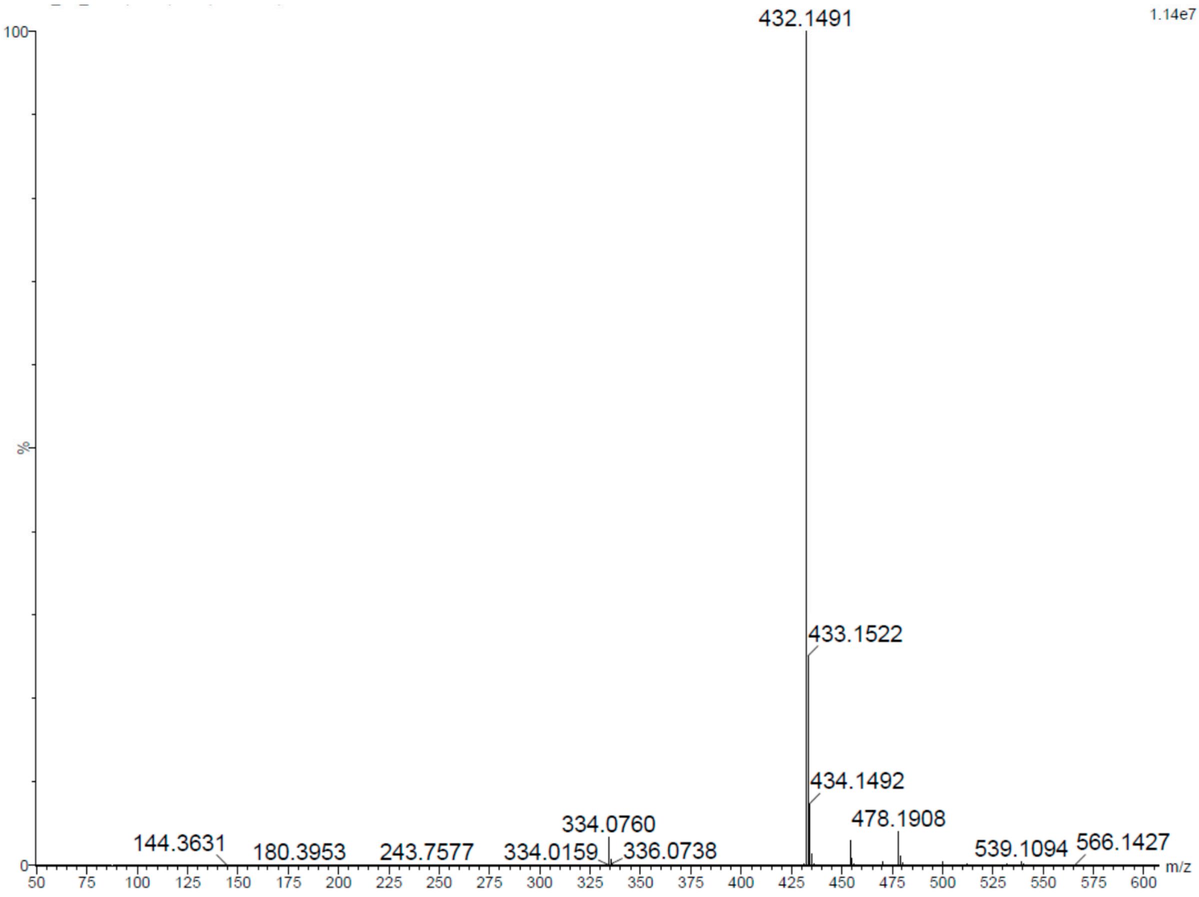
432.1491
1.14e7
100
%
0
433.1522
434.1492
478.1908
144.3631
180.3953
243.7577
334.0760
334.0159
336.0738
539.1094
566.1427
m/z
50
75
100
125
150
175
200
225
250
275
300
325
350
375
400
425
450
475
500
525
550
575
600

Product 3h

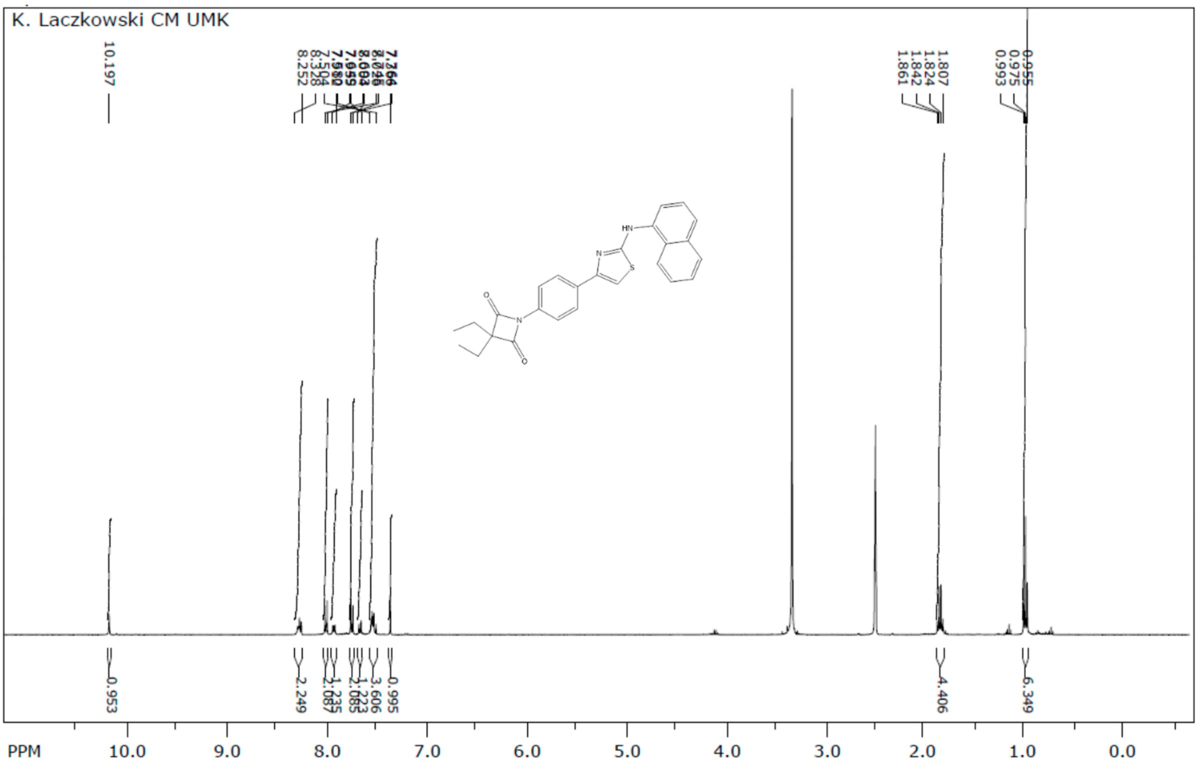
K. Laczkowski CM UMK
PPM
10.0
9.0
8.0
7.0
6.0
5.0
4.0
3.0
2.0
1.0
0.0

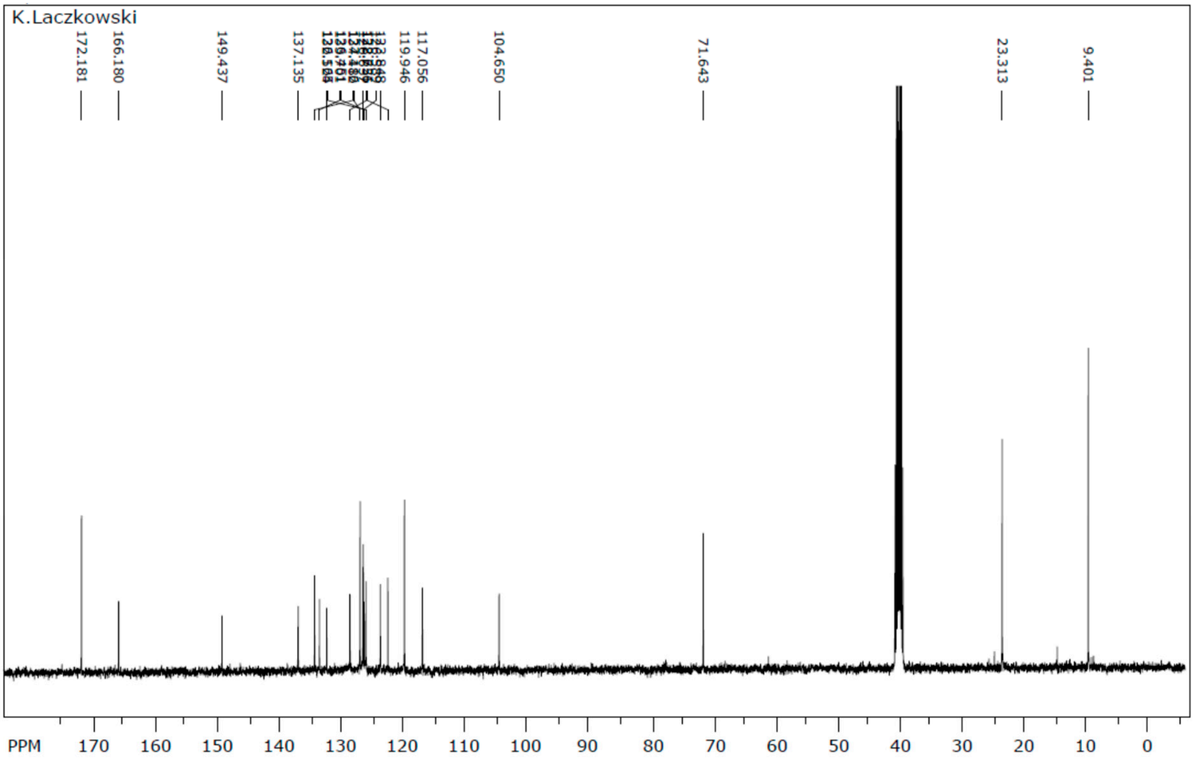
K.Laczkowski
172.181
166.180
149.437
137.135
119.946
117.056
104.650
71.643
23.313
9.401
PPM
170
160
150
140
130
120
110
100
90
80
70
60
50
40
30
20
10
0

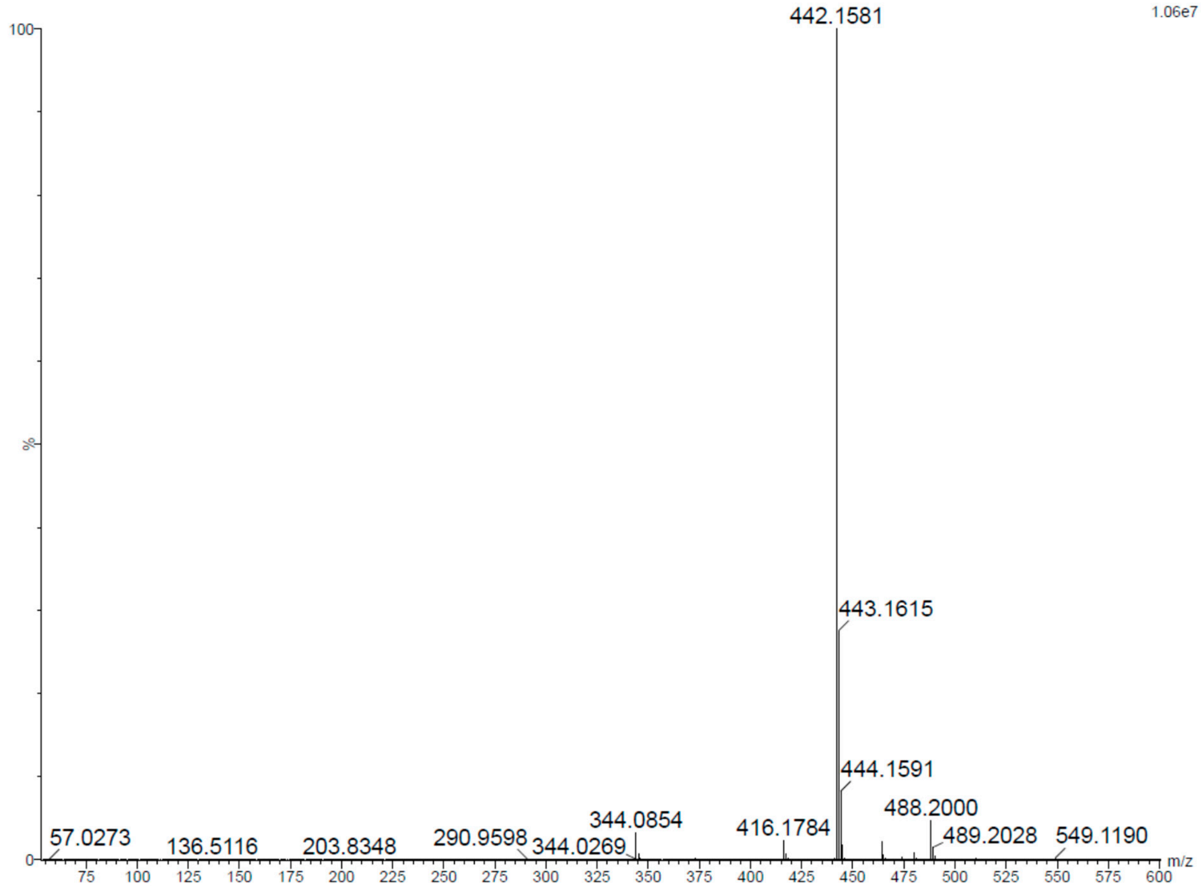
442.1581
1.06e7
443.1615
444.1591
488.2000
489.2028
549.1190
57.0273
136.5116
203.8348
290.9598
344.0269
344.0854
416.1784
%
m/z

Product 3i

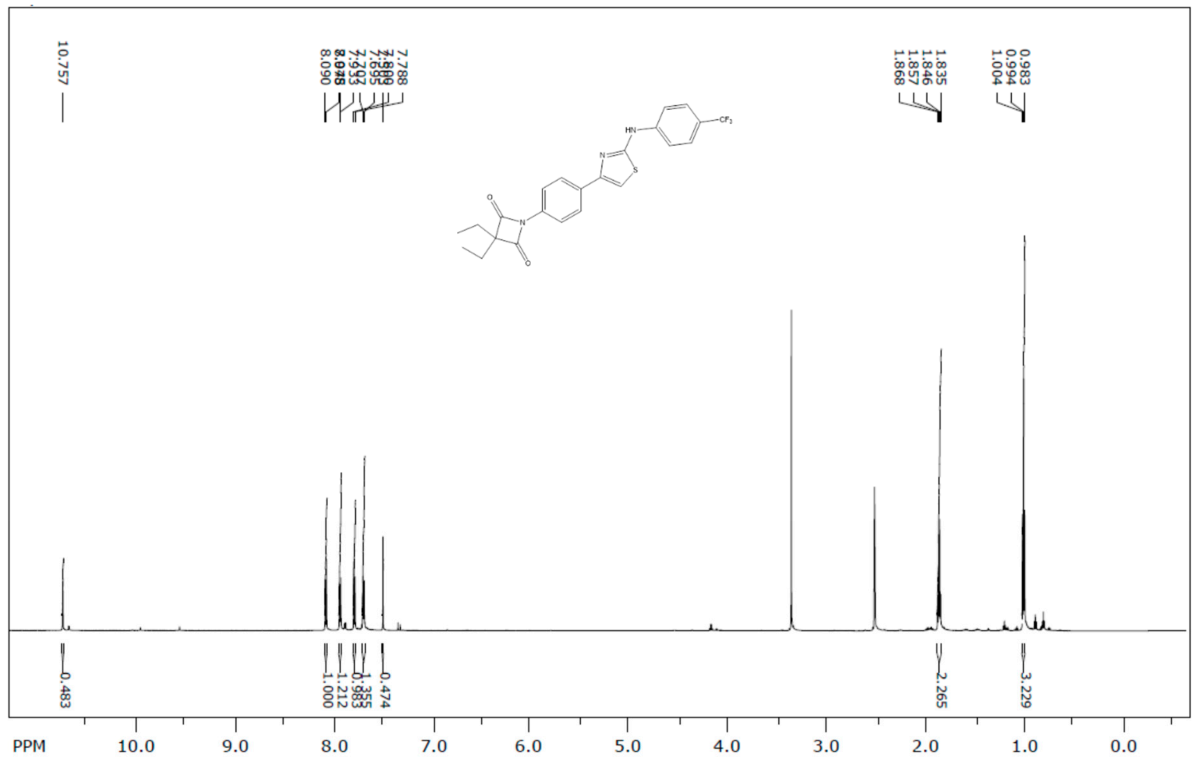
10.757
8.090
7.788
1.868
1.857
1.846
1.835
1.004
0.994
0.983
0.483
1.000
1.212
0.474
2.265
3.229
PPM

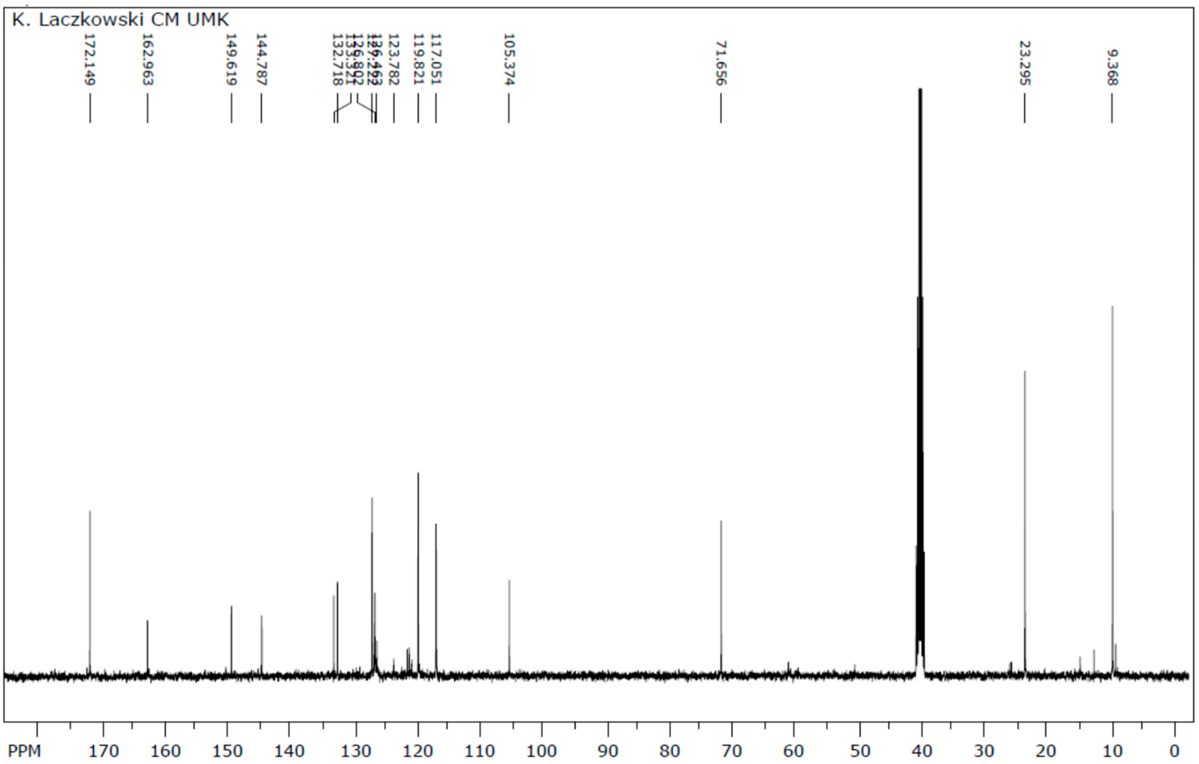
K. Laczkowski CM UMK
172.149
162.963
149.619
144.787
132.718
123.782
119.821
117.051
105.374
71.656
23.295
9.368
PPM
170 160 150 140 130 120 110 100 90 80 70 60 50 40 30 20 10 0

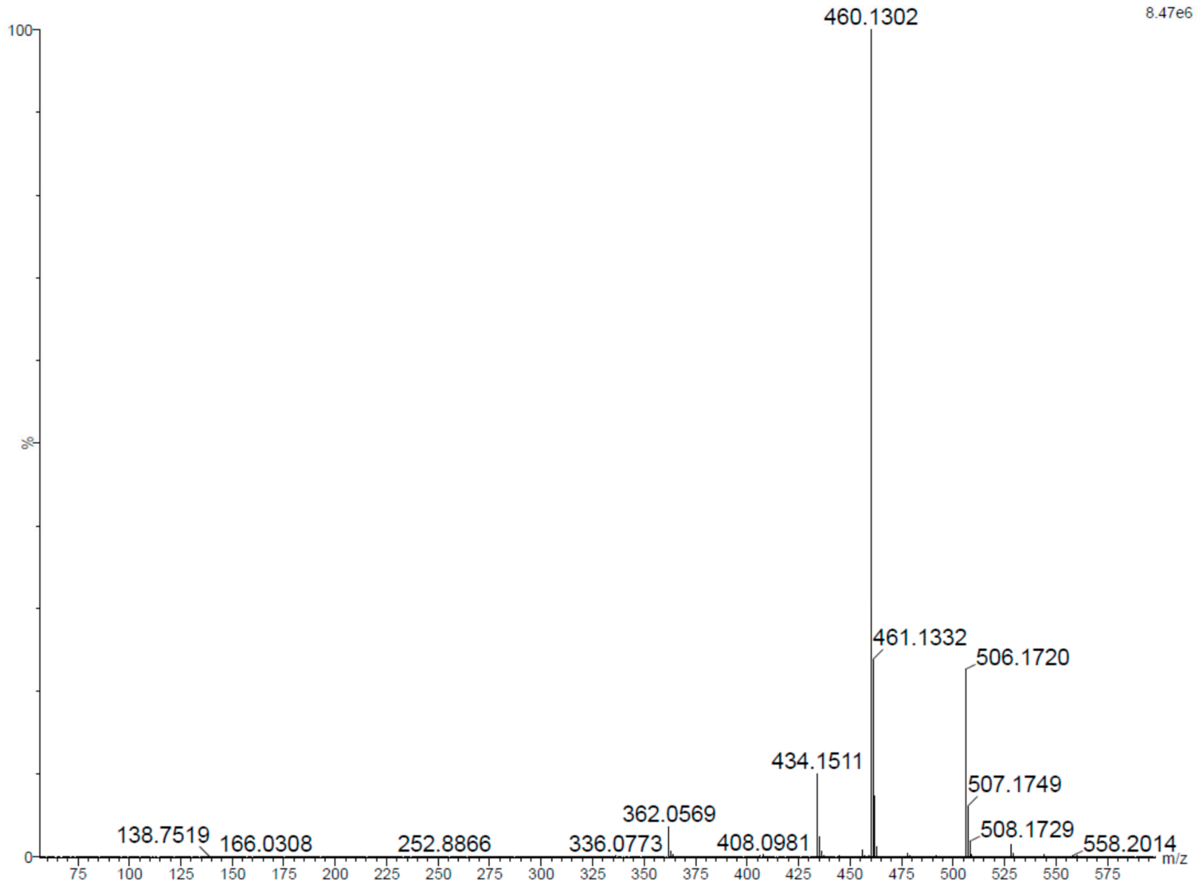
460.1302
8.47e6
100
%
0
461.1332
506.1720
434.1511
507.1749
362.0569
508.1729
138.7519
166.0308
252.8866
336.0773
408.0981
558.2014
m/z
75 100 125 150 175 200 225 250 275 300 325 350 375 400 425 450 475 500 525 550 575

Product 3j

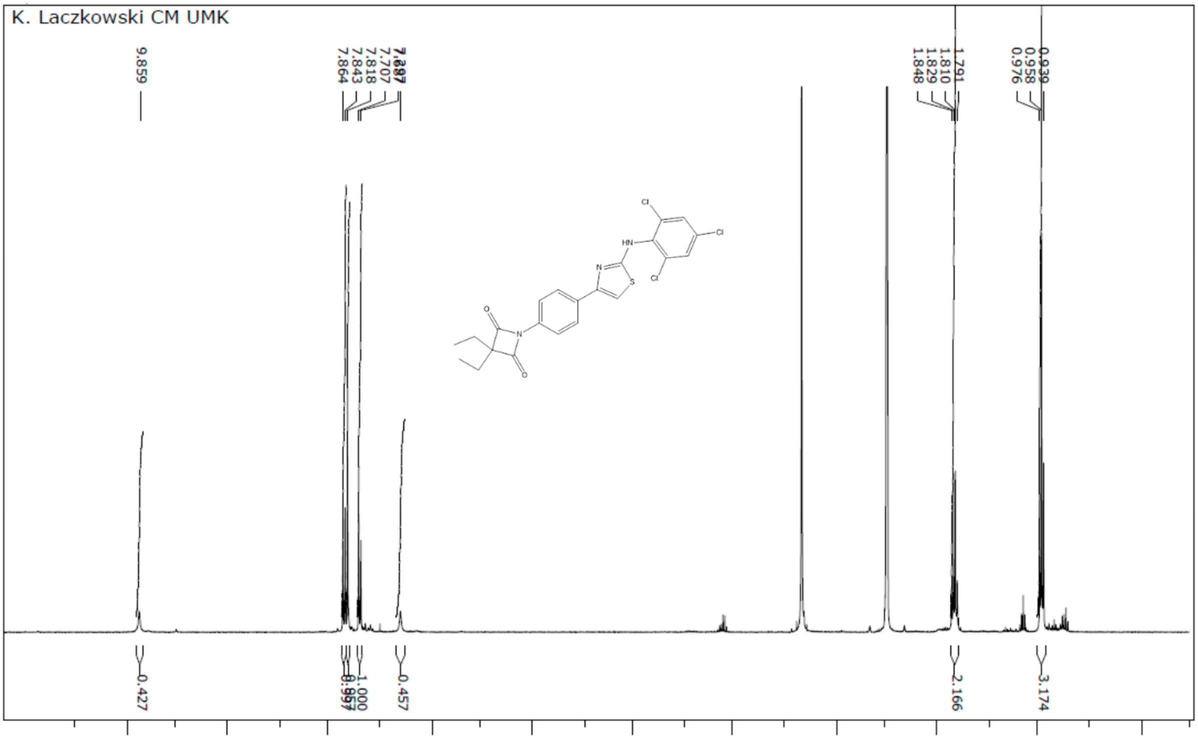
K. Laczkowski CM UMK
9.859
7.864
7.843
7.818
7.707
1.791
1.810
1.829
1.848
0.939
0.958
0.976
0.427
1.000
0.457
2.166
3.174
PPM
10.0
9.0
8.0
7.0
6.0
5.0
4.0
3.0
2.0
1.0
0.0
Cl
HN
N
S
O
N

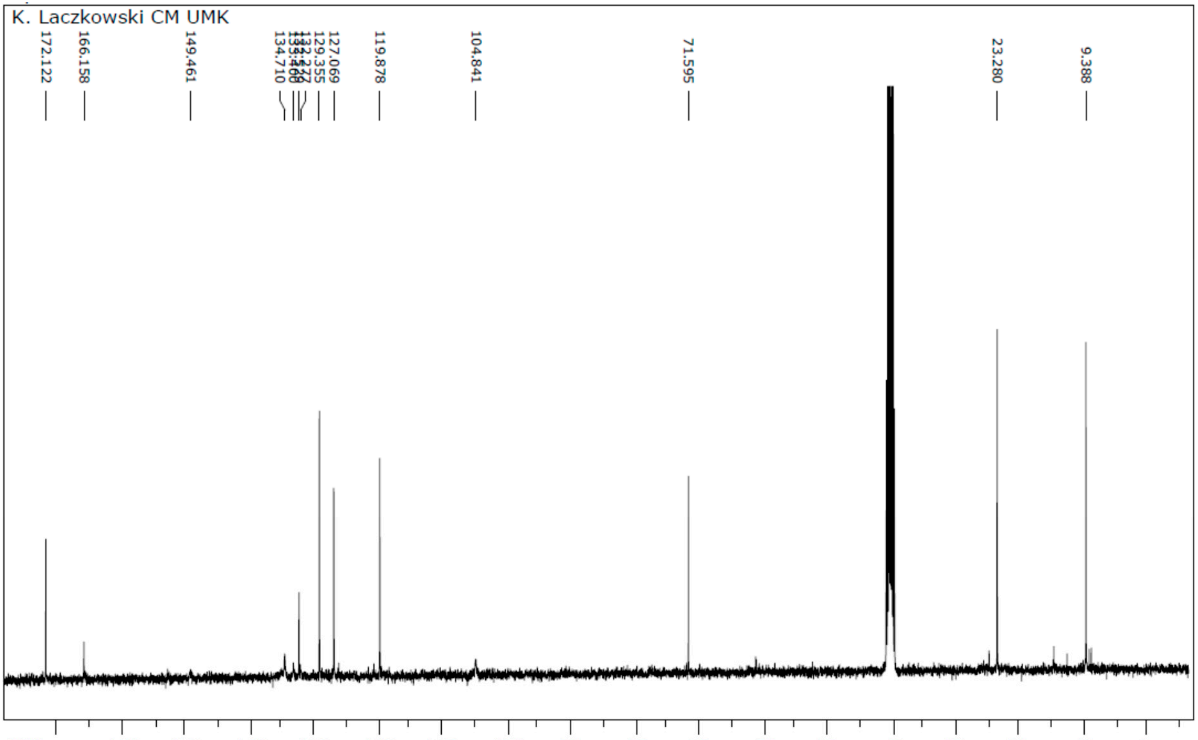
K. Laczkowski CM UMK
172.122
166.158
149.461
134.710
129.355
127.069
119.878
104.841
71.595
23.280
9.388
PPM
160
150
140
130
120
110
100
90
80
70
60
50
40
30
20
10
0

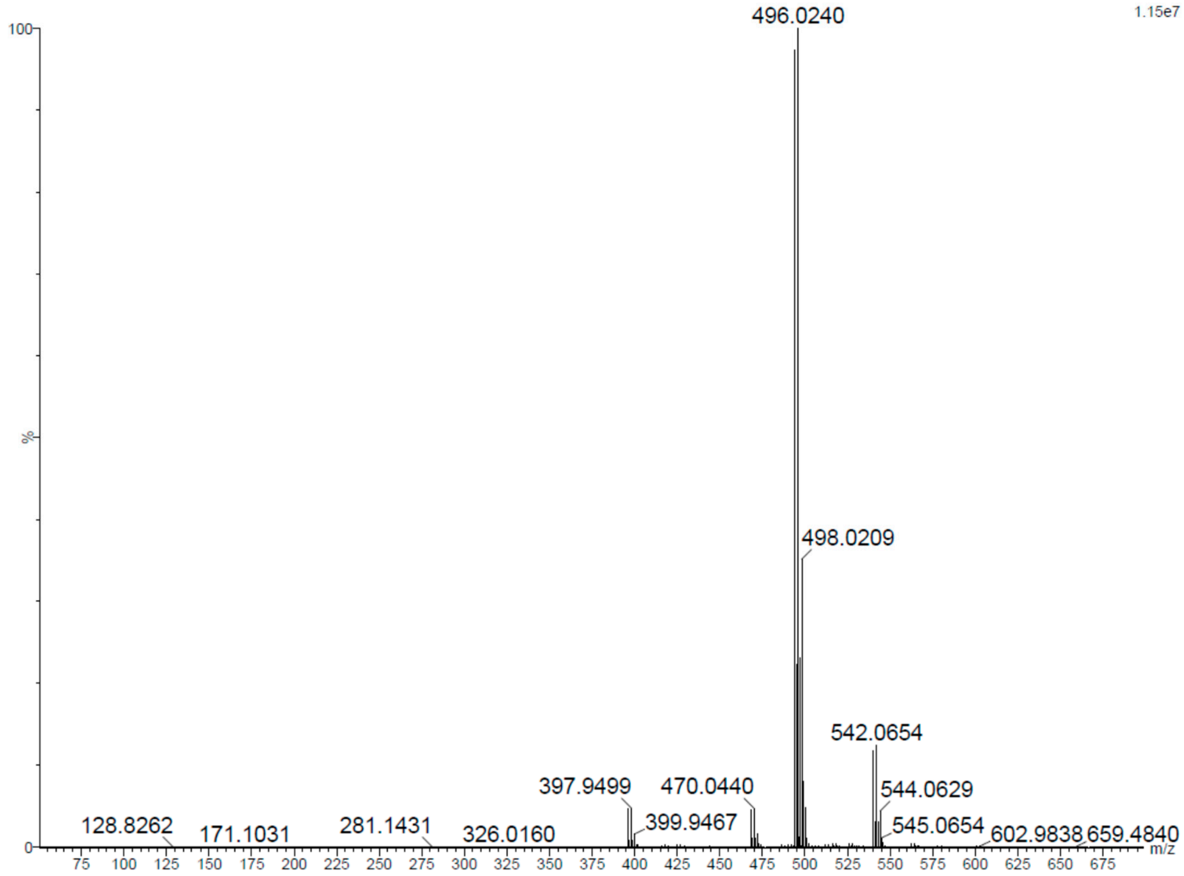
1.15e7
496.0240
498.0209
542.0654
544.0629
545.0654
397.9499
399.9467
470.0440
128.8262
171.1031
281.1431
326.0160
602.9838
659.4840
100
%
0
75
100
125
150
175
200
225
250
275
300
325
350
375
400
425
450
475
500
525
550
575
600
625
650
675
m/z
